# Metal-induced malformations in early Palaeozoic plankton are harbingers of mass extinction

**DOI:** 10.1038/ncomms8966

**Published:** 2015-08-25

**Authors:** Thijs R. A. Vandenbroucke, Poul Emsbo, Axel Munnecke, Nicolas Nuns, Ludovic Duponchel, Kevin Lepot, Melesio Quijada, Florentin Paris, Thomas Servais, Wolfgang Kiessling

**Affiliations:** 1 Unité Evolution, Ecologie et Paléontologie—UMR 8198, CNRS/Université de Lille—Sciences et Technologies, Avenue Paul Langevin, bâtiment SN5, 59655 Villeneuve d'Ascq, France; 2 Department of Geology and Soil Sciences (WE13), Ghent University, Krijgslaan 281/S8, 9000 Ghent, Belgium; 3 U.S. Geological Survey, Central Mineral and Environmental Resources Science Center, Denver Federal Center, Denver, Colorado 80225, USA; 4 Universität Erlangen-Nürnberg, GeoZentrum Nordbayern, Fachgruppe Paläoumwelt, Loewenichstrasse 28, D-91054 Erlangen, Germany; 5 IMMCL Chevreul, Université de Lille—Sciences et Technologies, Cité Scientifique, bâtiment C3, 59655 Villeneuve d'Ascq, France; 6 LASIR—UMR 8516, CNRS/Université de Lille—Sciences et Technologies, Cité Scientifique, bâtiment C5, 59655 Villeneuve d'Ascq, France; 7 Laboratoire d'Océanologie et de Géosciences—UMR 8187, CNRS/Université de Lille—Sciences et Technologies, Avenue Paul Langevin, bâtiment SN5, 59655 Villeneuve d'Ascq, France; 8 Géosciences Rennes—UMR 6118, CNRS/Université de Rennes 1, Campus de Beaulieu, 35042 Rennes, France; 9 Museum für Naturkunde, Leibniz Institute for Research on Evolution and Biodiversity at the Humboldt University Berlin, 10115 Berlin, Germany

## Abstract

Glacial episodes have been linked to Ordovician–Silurian extinction events, but cooling itself may not be solely responsible for these extinctions. Teratological (malformed) assemblages of fossil plankton that correlate precisely with the extinction events can help identify alternate drivers of extinction. Here we show that metal poisoning may have caused these aberrant morphologies during a late Silurian (Pridoli) event. Malformations coincide with a dramatic increase of metals (Fe, Mo, Pb, Mn and As) in the fossils and their host rocks. Metallic toxins are known to cause a teratological response in modern organisms, which is now routinely used as a proxy to assess oceanic metal contamination. Similarly, our study identifies metal-induced teratology as a deep-time, palaeobiological monitor of palaeo-ocean chemistry. The redox-sensitive character of enriched metals supports emerging ‘oceanic anoxic event' models. Our data suggest that spreading anoxia and redox cycling of harmful metals was a contributing kill mechanism during these devastating Ordovician–Silurian palaeobiological events.

## Abstract (web summary)

Metal toxicity is a primary source of abnormalities in aquatic organisms, and these have been used to evaluate anthropogenic heavy metal pollution. Here, the authors suggest that abnormalities in Silurian acritarchs were caused by heavy metal pollution corresponding to Early Palaeozoic extinction events.

## Graphical abstract

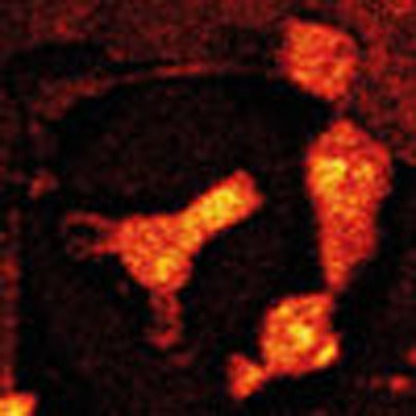

Ordovician–Silurian rocks record several dramatic, positive stable isotope excursions indicative of major disturbances of the oceanic carbon, oxygen and sulfur cycles^1^,^2^. The environmental changes linked to these disturbances had a devastating effect on marine life. One of these events (the end-Ordovician Hirnantian event) features among the ‘Big Five' Phanerozoic mass-extinction episodes^3^. Several other Ordovician–Silurian events are also marked by substantially elevated extinction rates among certain groups of marine taxa^4^,^5^,^6^, including the decimation of up to 95% of graptolite species during the Mulde event^5^ and 80% of conodonts during the Ireviken event^4^, along with losses in trilobites, brachiopods, corals, acritarchs, graptolites and chitinozoans. The Pridoli event that is studied here mainly affected corals, chitinozoans, graptolites and conodonts. Current causative models often revolve around the direct effects of changing palaeoclimate, such as cooling temperatures and habitat reduction, but do not fully integrate observed palaeontological, geological and geochemical shifts during these events^7^,^8^. Rooted in earlier models^9^, emerging geochemical and sedimentological evidence suggests that some of these disturbances are more directly linked to global oceanic anoxic events (OAEs) instead^10^,^11^,^12^. With the exception of some obvious links such as the drawdown of marine oxygen levels, however, the full set of interactions between OAEs originating in deep oceanic waters and mechanisms leading to extinctions across groups and palaeo-ecological niches on the continental shelves is still poorly understood. Elevated abundances of malformed (teratological) fossil microplankton (acritarchs and chitinozoans) mark the onset of the global, positive δ^13^C_carb_ excursions^13^, and coincide with the initiation of extinction^14^.

Whereas modern instances of teratology in aquatic micro- and macroorganisms have been linked to a variety of environmental stressors, the teratological effects of heavy metal pollution have been well established in the natural environment and laboratory experiments. Malformation in diatoms^15^,^16^, foraminifera^17^,^18^, flagellates^19^, echinoderms^20^, arthropods^21^ and fish^22^,^23^ is now routinely used as a powerful forensic tool to identify contamination of fresh, brackish and marine waters. Biomonitoring of ecotoxicity using eggs (for example, of copepods or cladocerans) typically focuses on hatching success and viability of embryos and neonates^24^. Nevertheless, malformation has also been observed in copepod egg^25^ and chrysophyte algae cyst morphologies^26^, respectively, triggered by diatom-enriched diets of the parent copepods, and Mn–Cd metal pollution of the environment of the algae that originally produced the cysts. In terms of affected functional morphology, these modern case studies involving cysts and eggs are strikingly similar to those of chitinozoans and acritarchs in the early Palaeozoic record. In the modern, such studies of resting stages are uncommon because the producing organism can be studied directly. As soft-bodied parent organisms are usually not preserved in the fossil record, we are obliged to use readily preserved egg cases or cysts to reconstruct the original oceanic conditions.

Here, in analogy with the modern, we test whether palaeo pollution of the marine environment by toxic metals might have also caused the Silurian teratologies. We explore the geochemical signal of chitinozoan assemblages at one of the events with elevated abundances of malformed specimens near the end of the Silurian. Chitinozoans are organic-walled microfossils (palynomorphs), 100–500 μm long, which probably represent fossil egg cases of marine zooplankton that lived in the shallow mixed layer of the Ordovician–Silurian oceans^27^. We show that their teratology coincides with metal enrichment in host rocks and fossils.

## Results

### Chitinozoan teratology

**Figure 1:**
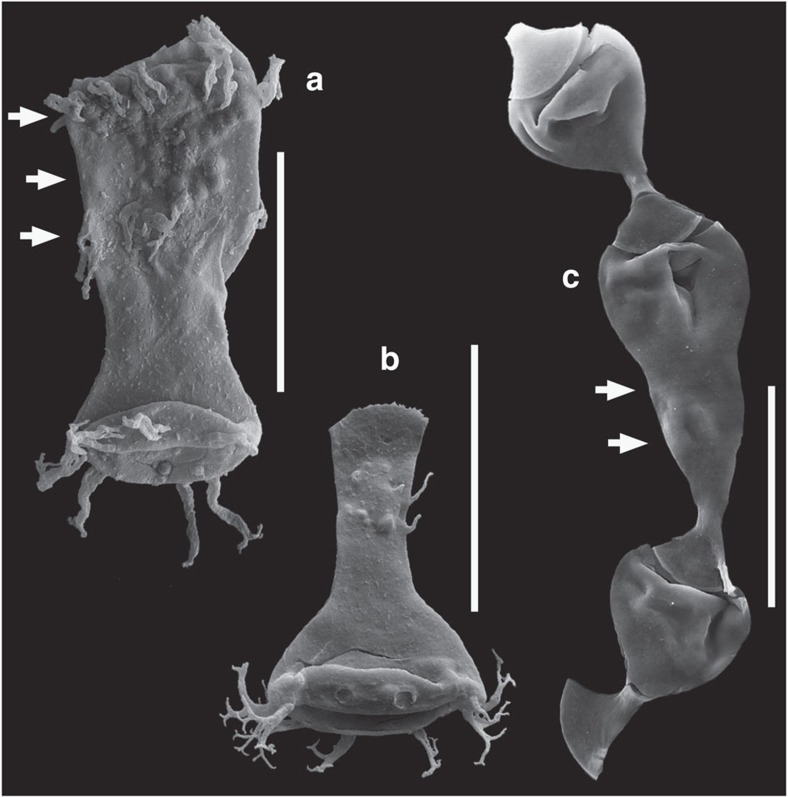
Teratological and normal chitinozoans from core 14 in the A1-61 well. (**a**) Teratological specimen of *Ancyrochitina* (sample 2,127.5 m). (**b**) Morphologically normal specimen of *Ancyrochitina* for comparison (sample 2,127.5 m). (**c**) Chain of three specimens of *Margachitina*, with an abnormal specimen in between two normal ones (sample 2,126.75 m). Scale bars, 100 μm. Arrows indicate abnormal growth features.

**Figure 2:**
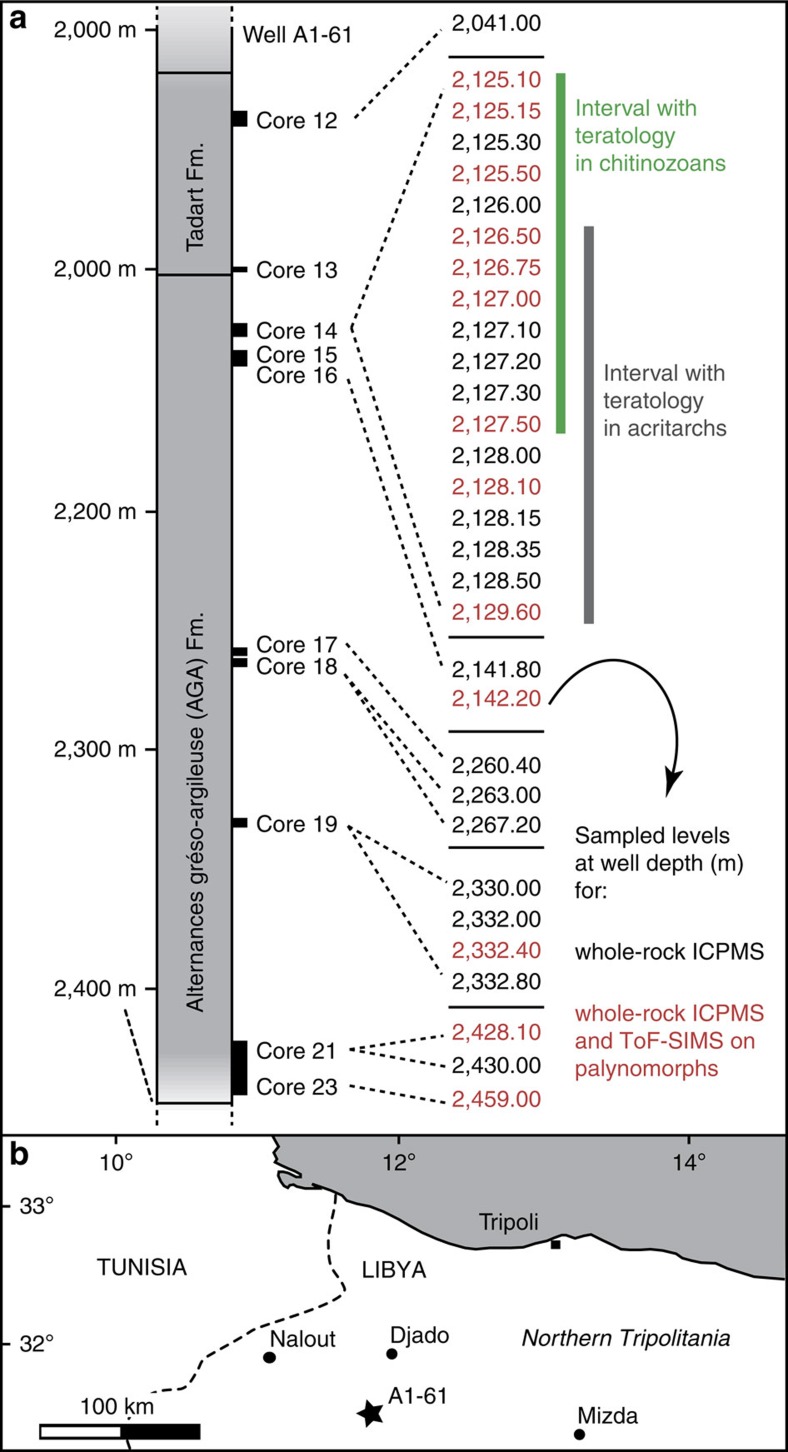
The studied well section. (**a**) A1-61 well section showing the cored intervals and analysed samples. The teratological interval for chitinozoans in core 14 has been extended downwards by 0.5 m compared with published data^29^ as we found additional malformed specimens at 2,127.5-m depth. The documented acritarch teratology from core 19 is from samples (2,333.8–2,333.7 m) that are outside of the series available to us for analyses^30^. (**b**) Map of well locality in Libya.

Malformed acritarchs (cysts of marine phytoplankton^28^) can be abundant during these ancient events. For instance, there are up to 20% of teratological specimens in the affected assemblages from the Hirnantian of Anticosti Island^13^,^14^. The documented proportions of malformed chitinozoans are much lower: assemblages with c. 0.1% of affected specimens are considered anomalous, markedly above estimated background values of 0.01–0.001% (ref. 29). Nevertheless, we focused on the analysis of chitinozoans (Fig. 1) because they are an order of magnitude larger in size than acritarchs and thus easier to manipulate and analyse. We apply a new combination of techniques to measure the metal content of individual palynomorph specimens from teratological assemblages using time-of-flight secondary ion mass spectrometry (ToF-SIMS) and the bulk rock that hosts them, using laser ablation-inductively coupled plasma-mass spectrometry (LA-ICP-MS; see Methods). We selected a mid-Pridoli (late Silurian) event for which a teratology anomaly in the palynomorphs has been well established^29^,^30^. The samples are from a well in the Libyan desert that cuts through the Mesozoic and Palaeozoic sequence of the Gondwanan platform (Fig. 2). The studied interval consists of a sequence of regularly alternating siltstones and shales, with thin oolithic-ferruginous beds near the top of the section. Samples were collected from a series of cored intervals (Fig. 2) that yield abundant, exquisitely preserved palynomorphs. In core 14, teratological assemblages of chitinozoans^29^ and acritarchs^30^ of mid-Pridoli age (*Margachitina elegans* biozone^31^) have been previously described, respectively, between 2,127.5 and 2,125 m, and 2,129.6 and 2,126.4 m. This narrow cored interval yielded multiple samples and assemblages where the frequency of teratological chitinozoan specimens was one or two orders of magnitude above background estimates^29^. Published counts exceed 0.1% of malformed specimens and even up to 1.3% of affected ancyrochitinids^29^. Many additional specimens have been recovered from the same levels in the current study (Supplementary Dataset 1). Any taphonomic causes for the teratological morphologies have been excluded beyond reasonable doubt^29^,^30^. The morphological deformities consist of significant defects, including wild growth of tissue, and the uncontrolled twinning of specimens (Fig. 1a), which are clearly outside of the range of intraspecific variability. These cannot be confused with regularly occurring ecophenotypes of the same species, which display a continuous spectrum of much more subtle morphological variability linked to the changes in ecology, as for instance documented in the length of dinocyst spines that varies with salinity^32^.

### Geochemistry

**Figure 3:**
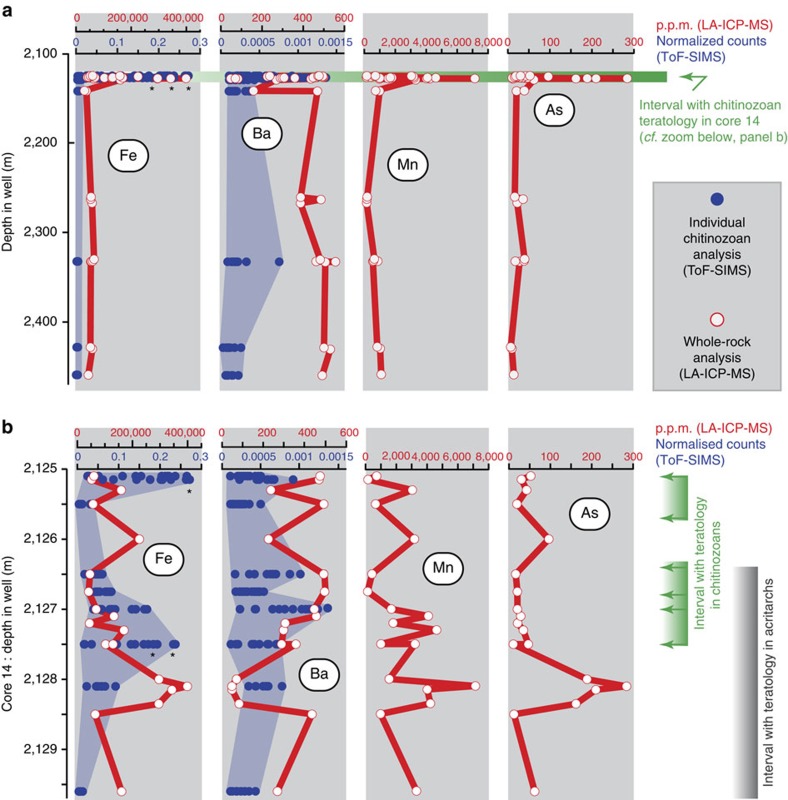
Geochemical signal of selected elements through well A1-61. (**a**) Whole-rock Fe, Mn and As data display clear peaks in the chitinozoan teratological interval, markedly above background values lower in the well (LA-ICP-MS, in p.p.m.). Analyses on individual chitinozoans exhibit peaks in certain elements (Fe and Ba) in the teratological interval (ToF-SIMS, semi-quantitative). Asterisks (*) indicate saturation at detector (that is, underestimated values). Elements (for example, As and Mn) subject to spectral interference and/or below detection limit of the ToF-SIMS analyses cannot be directly compared with the bulk rock. (**b**) Geochemical signals in the event horizon. Concentrations of whole-rock Fe, Mn, and As data, as well as Fe and Ba in individual chitinozoans are significantly above background. Green arrows mark samples with teratological chitinozoans.

**Figure 4:**
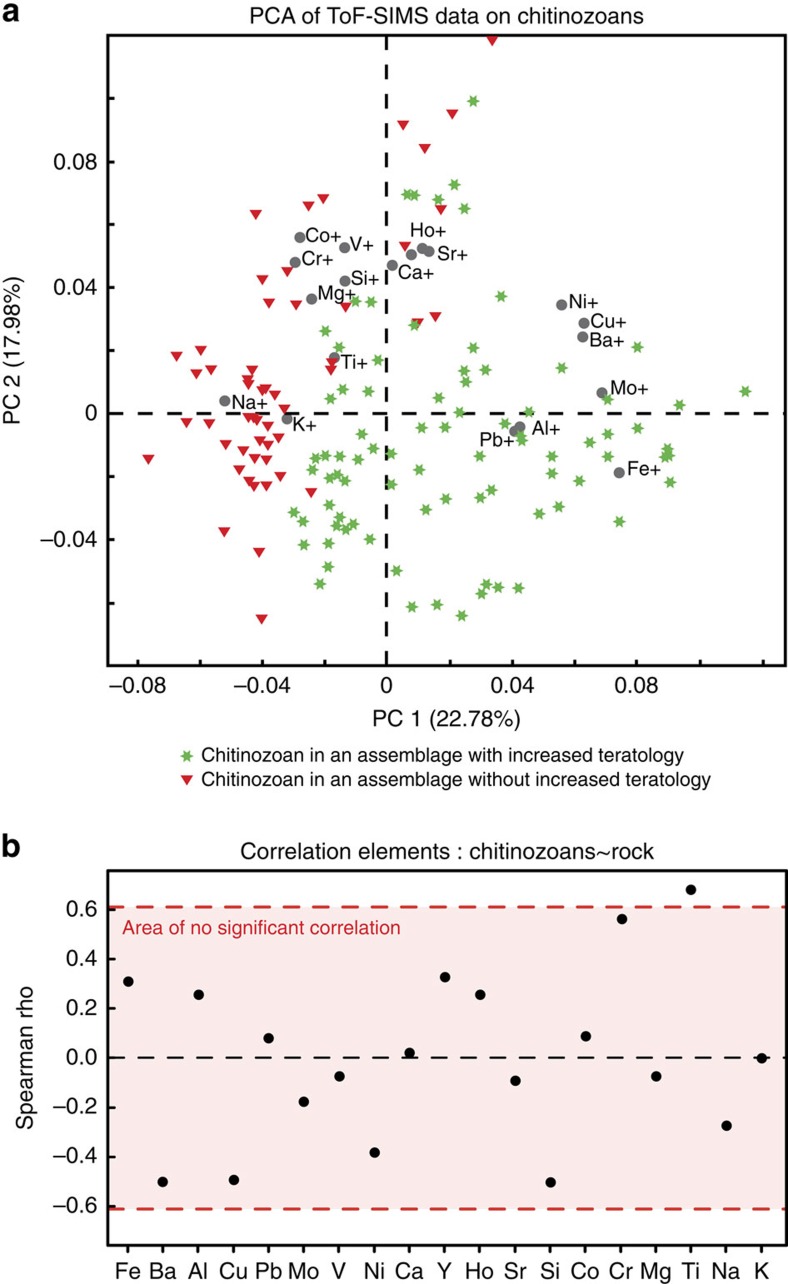
Statistical analyses of the geochemical data. (**a**) Principal component analysis (PCA) of ToF-SIMS data of chitinozoans indicates a clear separation of samples from within (green stars) and outside (red triangles) of their teratological interval, based on their geochemistry and mainly driven by elements such as Fe, Al, Cu, Ba, Mo and Pb. The PCA was carried out using the Eigenvector PLS toolbox (Eigenvector Research Inc., Wenatchee, WA, USA) in the MATLAB environment version 8.0 (The MathWorks, Natick, MA, USA). Data on 19 elements of 141 ToF-SIMS spectra (spread over 13 stratigraphical levels) were pre-scaled using auto-scaling^47^ (spectral variables are scaled to a zero mean and unit variance) as some of the spectral peaks of interest are relatively small. Three samples were extremely enriched in Fe and Al and these outliers were eliminated. (**b**) Correlation values between element concentrations in chitinozoans and whole-rock data. Measurements were averaged across 20-cm intervals of core and scaled to a mean of zero and unit variance before non-parametric correlation tests. Red lines indicate approximate correlation coefficients for a significant cross-correlation (*P*=0.05). Only Ti is significantly correlated between chitinozoans and rock samples. PC, principal component.

The metal content, particularly Fe, Cu, As, Al, Pb, Ba, Mo and Mn, of individual chitinozoans and/or bulk-rock samples increases markedly in the teratological interval (Fig. 3a). Fe, As and Mn concentrations in bulk-rock samples increase by an order of magnitude, for example, from c. 50,000 to c. 400,000 p.p.m. Fe, on cue with the appearance of oolitic-ferruginous beds in the stratigraphic section (Fig. 3b). Principal component analysis on the ToF-SIMS data clearly separates the lower samples from those of the teratological interval; this is due to elevated metal content of the chitinozoan specimens in assemblages with malformation, notably of Fe, Al, Mo and Ba (Fig. 4a). While the metal enrichment in the teratology horizon is evident both in individual chitinozoans and in bulk-rock chemistry, abundances of certain elements in bulk-rock samples are decoupled from those in the palynomorphs: for example, bulk-rock Ba contents are relatively constant, yet the chitinozoan Ba shows a significant increase in the teratological zone (Fig. 3a). In addition, at decimetre scale and notably within the horizon with malformations, specimen and bulk-rock chemistry is statistically decoupled for all elements as evidenced by time-series cross-correlation tests (Figs 3b and 4b). This decoupling demonstrates that the chemistry of chitinozoans is not a mere imprint of rock composition but may preserve the original chemistry of the organisms and thus reflect metal abundances in their aquatic environment. Relying exclusively on the chemical signature of the chitinozoans to represent original ocean chemistry may be subject to debate. However, the coincidence between malformations, a primary biological feature developed *in vivo*, and the increased metal contents in the organisms and sediments, in combination, is best explained by an increase in dissolved metals in the water column during the life of the organism and deposition of the sediments. We conclude that metal pollution, that is, the uptake of those metals during life and/or harmful conditions that developed in tandem with a rising metal content of the seawater, is the most likely explanation for the increase of teratology observed in these fossils.

## Discussion

**Figure 5:**
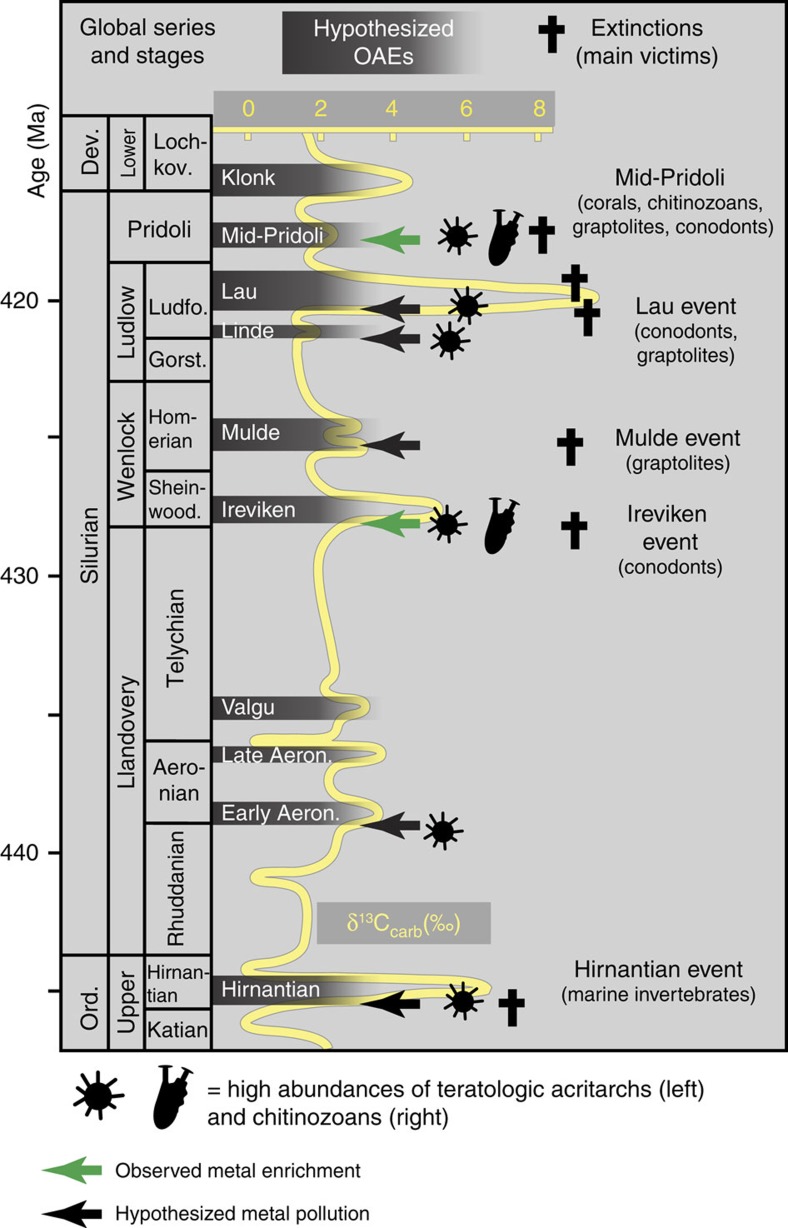
Distribution of potential OAEs in the uppermost Ordovician and Silurian. The position of the suggested OAEs is based on δ^13^C stratigraphy^34^. Key victims of the extinction events and the events with increased occurrences of teratology are highlighted^13^. Metal toxicity is observed in the Pridoli (this study) and implied during the Ireviken event, where metal enrichments in carbonates and phosphates in SE Sweden indicate anoxia^12^. For a full list of affected fauna see Kaljo *et al.*^33^ and Calner^6^. Ord., Ordovician; Dev., Devonian.

The mid-Pridoli teratology event correlates with faunal turnover^33^ and a positive δ^13^C excursion^34^ (Fig. 5). Early Palaeozoic bioevents were generally accompanied by large, positive δ^13^C_carb_ excursions. Consensus is converging that such signatures may reflect the burial and sequestration of isotopically light carbon on the deep seafloor during developing OAEs^35^. It is also well established that transitions to anoxic conditions in modern marine environments increase markedly the solubility of some metals in seawater^36^. Most significantly, suboxic conditions lead to the reductive dissolution of Fe–Mn oxyhydroxides, increasing the concentration of Fe and Mn in modern seawater by orders of magnitude. The concentration of some trace elements such as As, Mo and rare earth elements are directly controlled by absorptive scavenging and co-deposition with these oxyhydroxides; and as a consequence of reductive dissolution of oxyhydroxides, increase significantly in concentration in the seawater^37^,^38^,^39^. Moreover, transitions from oxic to anoxic oceanic conditions can trigger massive shifts in the cycling of Fe–Mn oxyhydroxides sequestered in sub-seafloor sediments, thereby triggering a benthic flux of Fe, Mn and associated trace elements from the sediments into the anoxic seawater^37^,^38^,^40^. Other elements, such as Ba, are governed by linked processes such as the reduction of sulfate in anoxic sediments^41^. We posit that the observed metal enrichments and teratology during this Pridoli event reflects the encroachment of metal-enriched, oxygen-depleted oceanic waters into the oxic environments of the continental shelves. As this redox front moved into shallow water, the toxic mix of elements was introduced into the shelf ecosystems teeming with Silurian life. Consequently, the metals were re-oxidized and precipitated, each at their own pace (Supplementary Dataset 1), at the redox interface between the anoxic water mass and local oxic surface waters. This scenario accounts for the accumulation of redox-sensitive metals in both the sediments and fossils and explains the teratology of organisms during the Pridoli event. This interpretation is supported by similar enrichments of redox-sensitive metals through the early Wenlock Ireviken event^12^, by the enrichment of rare earth elements in widespread phosphorites across Laurentia^42^, and by the concurrence of ironstones with Silurian C-isotope excursions in the Appalachian Basin (USA). The US sections display consistent patterns of red, green and black marine sedimentary rocks, indicative of increasingly anoxic conditions^10^. In this model, the harmful effects of spreading dead zones^43^, decreasing oxygen and increasing metal concentrations are intricately entangled. For instance, a study^44^ of the seasonally anoxic Chesapeake Bay links the sudden occurrence of malformed foraminifer *Ammonia* to the onset of anoxic conditions in the 1970s. However, it is also known that these seasonal anoxic conditions triggered the prompt release of redox-sensitive metals from the sediments into the water column^40^. In our Silurian data, metal-enriched malformed assemblages occur at the onset of the carbon isotope excursions, while hypoxia was starting to spread (and before it peaks later, indicated by the deposition of black shales^10^). This suggests that a direct toxic effect of metal enrichment contributed to the most plausible mechanism for the deformities in the organisms. Other environmental stressors that are known to cause malformation in the modern include changes in light intensity, ultraviolet radiation, predation, salinity and pH, and could have coincided with spreading anoxic waters, but there is no empirical evidence for such changes in the event interval.

The metal composition of marine sedimentary rocks is a fundamental parameter used to interpret the chemical evolution of the Earth's atmosphere and hydrosphere. Yet, generally, uncertainty remains regarding the primary metal abundances in seawater, pathways of metal accumulation and post-depositional modification of metal abundances in sedimentary rocks. As demonstrated in modern marine environments, our results suggest that metal-induced teratology of fossil plankton may serve as an independent proxy for monitoring changes in the metal concentrations of the shallow palaeo-ocean. This new proxy enables us here to reduce these uncertainties and supports the interpretation that the sedimentary chemistry reflects changing oceanic metal compositions during OAEs. As such, this proxy has the potential to help unravel the complexities inherent to sedimentary geochemistry, and may be a tool to evaluate other instances of marine metal variation through the geological record.

The co-occurrence between mid-Pridoli teratology and the onset of extinction suggests that metal contamination may have played a direct role in the biological crisis at large. More generally, the recurring temporal match between Ordovician–Silurian teratology events and extinction events^13^ raises the prospect that toxic metal contamination may be a previously unrecognized contributing agent to many, if not all, of these bioevents (Fig. 5). In sections where such data exist, teratological phytoplankton precede or co-occur with the earliest phases of these major extinction events. Taken at face value, for example, Hirnantian acritarch malformation in the Lousy Cove Member (member 6) of the Ellis Bay Formation coincides with the onset of the ‘phytoplankton crisis' on Anticosti Island (Canada) but precedes the major macrofauna extinctions in the overlying Laframboise Member (member 7) (refs 14, 45). On Gotland (Sweden), increased acritarch malformation occurs around a series of marker beds^46^, identifying an interval that starts below the strongest extinctions of conodonts during the early Ireviken event^4^. Although the patterns and exact relative timing of teratology and extinction require confirmation, the available data suggest that malformed palynomorphs could be the proverbial ‘canaries in the coal mines', that is, the first indicators of Palaeozoic extinctions.

Metal toxicity, and its fossilized *in vivo* expressions, could provide the ‘missing link' between extinction of faunas on the shelf, and widespread ocean anoxia. As part of a series of complex systemic interactions accompanying these shifts in ocean conditions, the redox cycling of metals may identify the early phase of the kill mechanisms that culminated in these catastrophic events. Although OAEs typically entail rapid climatic change, our data suggest that the proposed mechanisms of these early Palaeozoic mass extinctions were previously too simply linked to global cooling, invoking thermal stress and habitat reduction. Ultimately, metal-induced teratology might become a new forensic tool to identify original oceanic geochemical signatures and may help unravel the biologic and geochemical systematics that define these extraordinary periods of Earth history.

## Methods

### Material

Samples were studied from the 3,176 m A1-61 borehole in Ral-el-Dorah in Libya that cuts through the Mesozoic, Devonian, Silurian and into the Upper Ordovician sequence. The samples were collected from infrequently cored Silurian intervals. The original biostratigraphic purpose of the well explains why not every iron oolite bed in core 14 was sampled systematically, as oolites do not normally yield age-diagnostic microfossils (Fig. 2). Our samples 2,127 and 2,125.1 are direct subsamples from those of Jaglin and Paris^29^. Note that the teratological interval for chitinozoans in core 14 has been extended downwards by 0.5 m (Fig. 2) compared with published range^29^ as we found additional malformed specimens at 2,127.5 m (Supplementary Dataset 1).

### Palynology

Thirteen samples from A1-61 were dissolved for palynology. The core chips were broken into 0.5-cm pieces and decarbonated using 34% HCl. They were then agitated with c. 200 ml 48% HF over c. 24 h. Any newly formed fluor silicates were removed using a second 17% HCl treatment, over 32 h and at 60 °C. Samples were neutralized and filtered at 51 μm. Individual palynomorphs were handpicked from the organic residue (>51 μm) using a stereomicroscope at × 50–150 magnification, and transferred onto glass slides with Cu-tape and microscope grids, which were then used in ToF-SIMS analyses. Scanning electron microscope positioning of specimens versus reference grid was used to identify the specimens in the ToF-SIMS.

### Time-of-flight secondary ion mass spectrometry

Individual microfossils from 13 samples from A1-61 were analysed using ToF-SIMS. ToF-SIMS spectra measurements were carried out using a TOF.SIMS 5 instrument (ION-TOF GmbH, Germany). This instrument is equipped with a Bi liquid metal ion gun. Pulsed Bi_3_^+^ primary ions have been predominantly used for analysis (25 keV, 0.3 pA). Analyses were performed semi-quantitatively using two settings: ‘mapping mode' (500 × 500-μm^2^ grids, and the acquired images were 512 × 512 pixels, which has the advantage of simultaneously analysing multiple specimens) and ‘focused beam mode' (focussing the Bi-pulsed beam on a 5 × 5-μm^2^ grid and in doing so removing surface layers from specimens). Both ‘mapping' and ‘focused beam' acquisitions were performed in high-current bunched mode. The primary Bi_3_^+^ ion current measured in the Faraday cup was 0.4 pA. In the mapping-mode experimental conditions, we obtained a lateral resolution of c. 1 μm. Mass resolution calculated at *m*/*z*=56 for Fe^+^ was >4,000. The primary ion dose density (PIDD, calculated after 10 shots per pixel) was 3 × 10^11^ ions per cm^2^, indicative of static analytical conditions. In the focused beam mode, the mass resolution calculated at *m*/*z*=56 for Fe^+^ remained >4,000. The primary ion dose density was 2 × 10^15^ ions per cm^2^ (non-static). The focused beam mode allows for a substantial reduction of modern contaminants (for example, PDMS, see Supplementary Fig. 1). The data from the 170 focused beam analyses (including 141 on chitinozoans) are those presented in the main paper (Supplementary Datasets 1 and 2). Measurements were accumulated over 133–200 s. The mapping-mode data are deposited as Supplementary Datasets 1 and 2, and mirror the trends of the focused beam-mode data. These ToF-SIMS maps also illustrate a uniform distribution of the key elements through the palynomorphs, excluding mineral origin (Supplementary Fig. 2).

Owing to the exploratory nature of study, the mapping-mode analyses used two different source modes, Bi^+^ and Bi_3_^+^. These are clearly separated in the Supplementary Dataset 1. For the ‘focused beam-mode' analyses, only the Bi_3_^+^ source was used (this is the data in Figs 3 and 4). The data from the ‘Bi_3_^+^ mapping mode' clearly displays the same trend as for the ‘focused beam' mode, that is, a clear increase in metals in the samples with teratological assemblages, compared with the other samples lower in the section (this is the bulk of the samples in Supplementary Dataset 1). For the ‘Bi^+^ mapping mode', this is somewhat less obvious (only three samples were analysed due to a markedly weaker signal), however, increases are observed for elements such as Fe, Al and Mo, in the chitinozoans of the two teratological samples analysed in Bi^+^ mode.

Sixty-six ion peaks were originally selected in the spectra. Twenty-one remained after eliminating those with potential overlap (for example, between peaks of Mn and ^54^FeH^+^, Ga^+^ and CHFe^+^) and those below the background noise threshold. We retained data for 19 ions (excluding H^+^ and C^+^) from a total number of 534 specimens of chitinozoans, acritarchs, scolecodonts (jaws of polychaete annelids), spores and graptolite fragments (organic-walled macro-zooplankton) that were analysed in mapping mode (Supplementary Dataset 2); a further 170 analyses, focussing on chitinozoans, were conducted in focused beam mode.

### Laser ablation-inductively coupled plasma-mass spectrometry

Major and trace element abundances for 32 bulk-sediment samples were determined by LA-ICP-MS at the USGS (Supplementary Dataset 3). Analyses are within 15% of accepted values of USGS reference materials, duplicate analyses of selected samples by inductively coupled plasma-atomic emission spectrometry and inductively coupled plasma-mass spectrometry (ICP-AES-MS) sodium peroxide sinter (SGS Mineral Services, Toronto) and in-house ICP-AES-MS sinter methods. Replicate analyses were <3% RSD.

### Cross-correlation

Chemical measurements of whole rocks and fossils were partitioned into 20-cm regular-spaced intervals between 2,125.1 and 2,459 m. Chemical measurements of each element were averaged within each interval and empty intervals (no measurements) were omitted from each time series. Averaged values were standardized to unit variance and a mean of zero before performing cross-correlation tests. Reported correlation coefficients are without temporal lag and non-parametric (Spearman's Rho).

## Additional information

**How to cite this article:** Vandenbroucke, T. R. A. *et al.* Metal-induced malformations in early Palaeozoic plankton are harbingers of mass extinction. *Nat. Commun.* 6:7966 doi: 10.1038/ncomms8966 (2015).

## Supplementary Material

Supplementary Information Supplementary Figures 1-2

Supplementary Data 1 Monovariate plots per element, where paired SIMS and ICPMS data are available (.pdf).

Supplementary Data 2 ToF-SIMS data matrices (.xlsx). Tab 1: focused beam mode data. Tab 2: mapping mode data.

Supplementary Data 3 LA-ICP-MS whole rock data (.xlsx).
