# Supplementary material for: Metal-induced malformations in early Palaeozoic plankton are harbingers of mass extinction
Source: Nat Commun. 2015 Aug 25;6:7966. doi: 10.1038/ncomms8966 (PMC4560756; doi:10.1038/ncomms8966)
Supplement: Supplementary Information — Supplementary Figures 1-2 [file ncomms8966-s1.pdf]

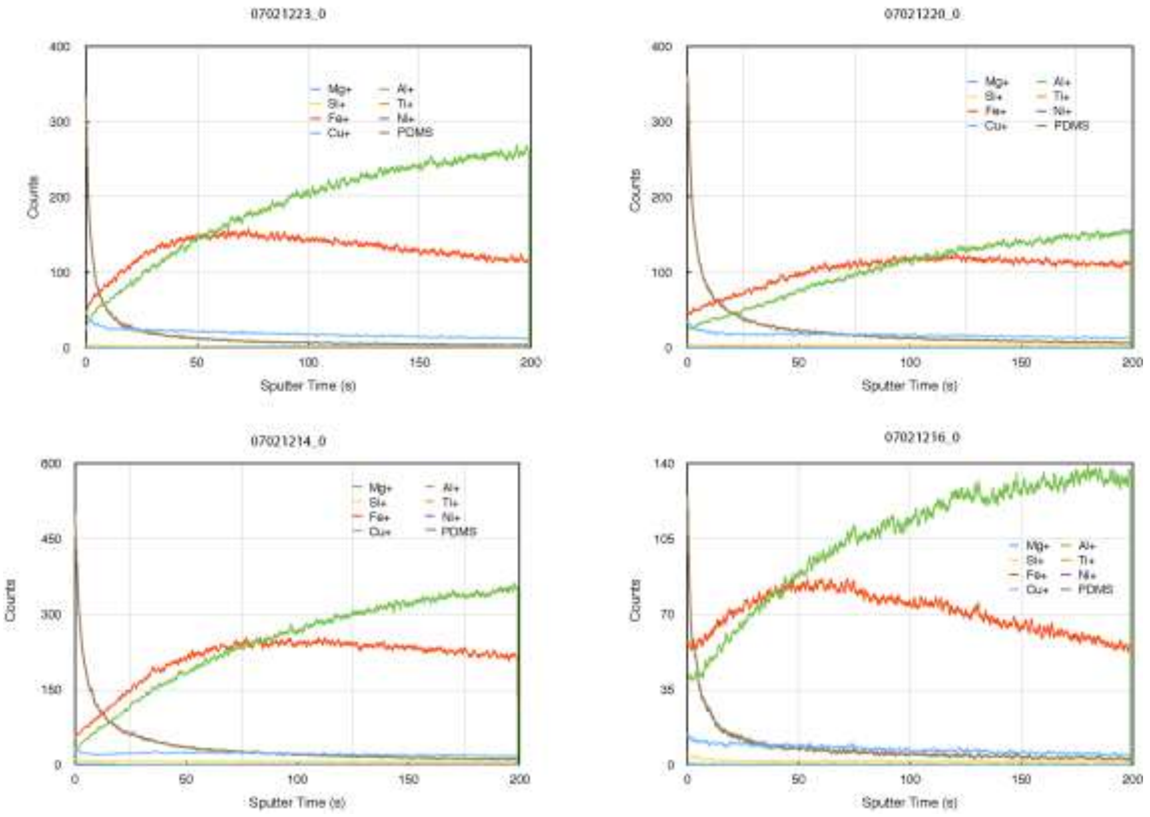

Supplementary Figure 1 | Progress plot of a few representative ‘focused beam’ ToF-SIMS analyses (0-200 seconds) from sample 2126.75m, where over time, the amount of modern contaminants (Polydimethylsiloxane - PDMS) notably drops, and the amount of registered counts of the metals of interest (Fe, Al) increases. For the plots in Figures 3 and 4 (and Supplementary Datasets 1 and 2), ‘focused beam’ counts were accumulated from between 133 to 200 seconds into the analyses, i.e., when the drop of modern pollutants and increase of original elements had stabilized.

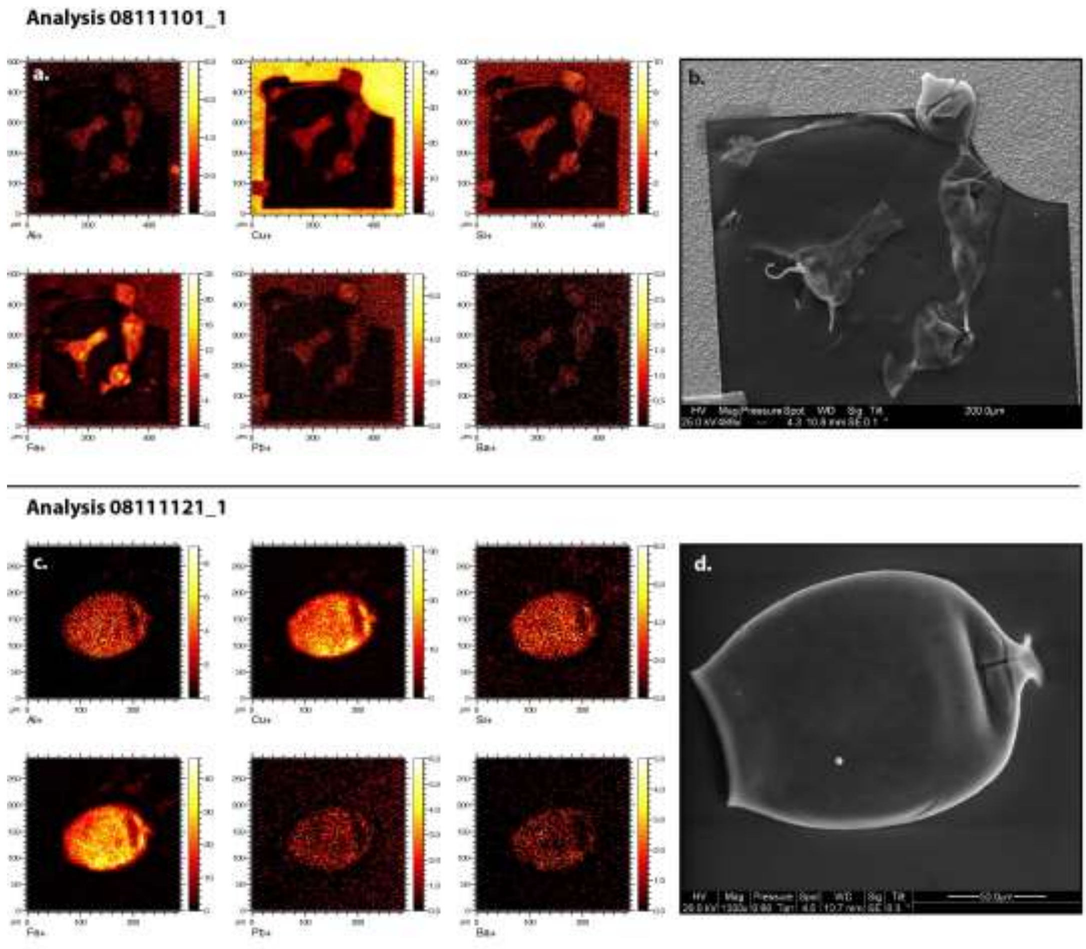

Supplementary Figure 2 │ Additional analyses illustrating the original character of the chitinozoan geochemistry. Shown are ToF-SIMS maps (500x500 µm grids) for several specimens of sample 2126.75. The metal signal, used in this paper, emerges uniformly from the chitinozoan specimens, and not from specific zones of the fossils. The opposite might have suggested the signal emerged from crystal growth. Tiny pyrite crystals (small white squares on the SEM images), as observed in some specimens such as the *Ancyrochitina* and *Urnochitina* specimens shown here, do not correlate with the Fe signal (see ToF-SIMS maps), indicating that sputtering of pyrite is low compared to organic matter and is not the source of the metal signals detected. Any heterogeneity in the element distribution is due to the relief of the specimens, causing certain areas to be shielded from the Bi_3^+ beam, which hits the specimens at an angle. **(a-b)** Analysis 08111101_1; slide ‘teratology’, specimen 39 (*Ancyrochitina* sp., left) and specimen 38 (right, a chain of 3 margachitinids, with one teratologic specimen in between two normal ones; cf. Fig. 2). **(a)** selected ToF-SIMS element maps. **(b)** SEM picture of same zone, post-analysis, Au coated specimen for improved imaging. The background grid high in Cu is a

microscope / SEM sample support grid that enables tracking of the specimens between SEM identification and ToF-SIMS analyses. **(c-d)** Analysis 08111121_1; slide ‘1’, specimen 10 (*Urnochitina urna*). **(c)** selected ToF-SIMS element maps. **(d)** SEM picture of same zone, pre-analysis, uncoated specimen.
