# Supplementary material for: Metal-induced malformations in early Palaeozoic plankton are harbingers of mass extinction
Source: Nat Commun. 2015 Aug 25;6:7966. doi: 10.1038/ncomms8966 (PMC4560756; doi:10.1038/ncomms8966)
Supplement: Supplementary Data 1 — Monovariate plots per element, where paired SIMS and ICPMS data are available (.pdf). [file ncomms8966-s2.pdf]

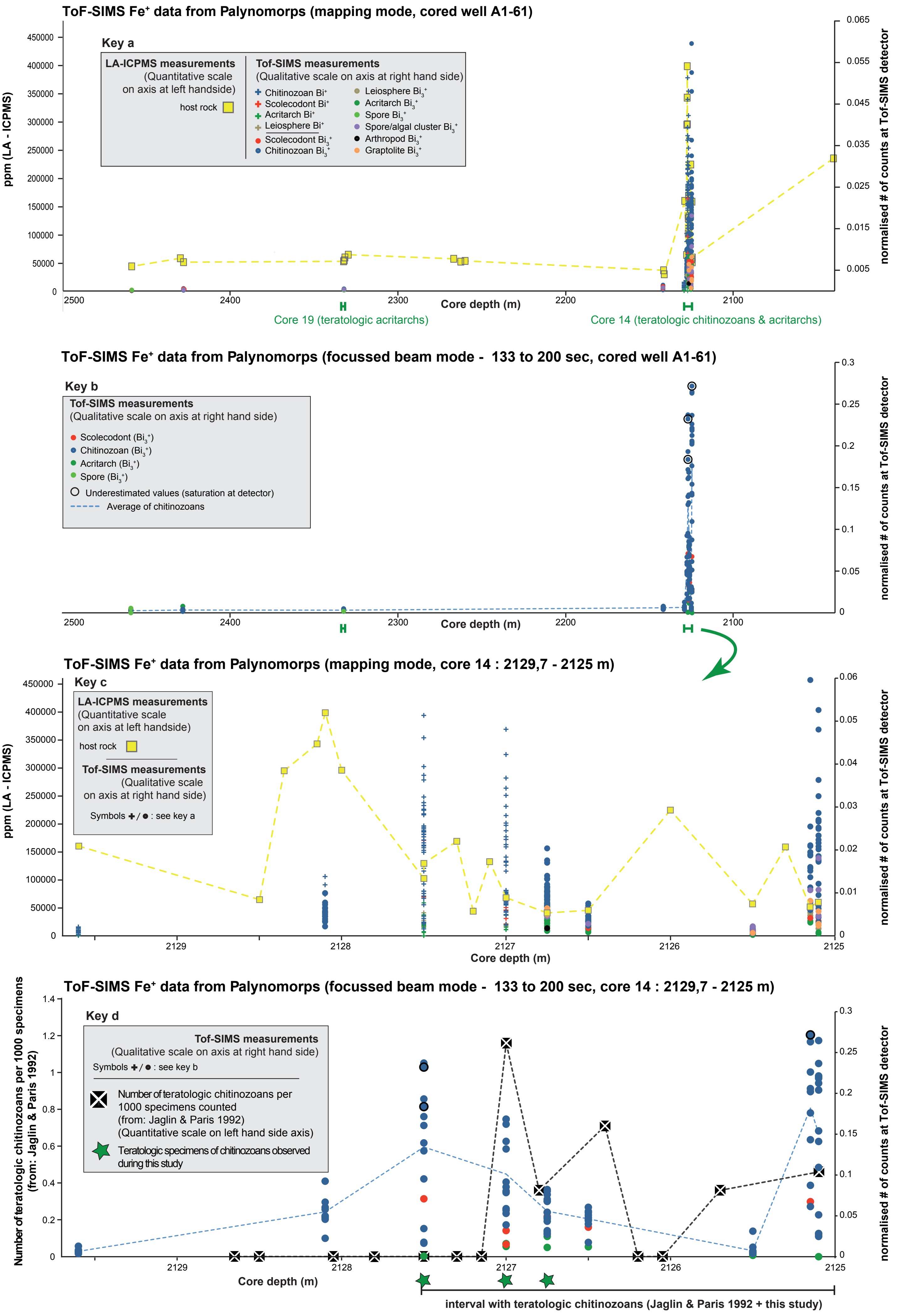

ToF-SIMS Fe+ data from Palynomorps (mapping mode, cored well A1-61)
Key a
LA-ICPMS measurements
(Quantitative scale on axis at left handside)
host rock
Tof-SIMS measurements
(Qualitative scale on axis at right hand side)
Chitinozoan Bi+
Scolecodont Bi+
Acritarch Bi+
Leiosphere Bi+
Scolecodont Bi3+
Chitinozoan Bi3+
Leiosphere Bi3+
Acritarch Bi3+
Spore Bi3+
Spore/algal cluster Bi3+
Arthropod Bi3+
Graptolite Bi3+
ppm (LA - ICPMS)
normalised # of counts at Tof-SIMS detector
Core depth (m)
Core 19 (teratologic acritarchs)
Core 14 (teratologic chitinozoans & acritarchs)
ToF-SIMS Fe+ data from Palynomorps (focussed beam mode - 133 to 200 sec, cored well A1-61)
Key b
Tof-SIMS measurements
(Qualitative scale on axis at right hand side)
Scolecodont (Bi3+)
Chitinozoan (Bi3+)
Acritarch (Bi3+)
Spore (Bi3+)
Underestimated values (saturation at detector)
Average of chitinozoans
ToF-SIMS Fe+ data from Palynomorps (mapping mode, core 14 : 2129,7 - 2125 m)
Key c
LA-ICPMS measurements
(Quantitative scale on axis at left handside)
host rock
Tof-SIMS measurements
(Qualitative scale on axis at right hand side)
Symbols ✚/● : see key a
ToF-SIMS Fe+ data from Palynomorps (focussed beam mode - 133 to 200 sec, core 14 : 2129,7 - 2125 m)
Key d
Tof-SIMS measurements
(Qualitative scale on axis at right hand side)
Symbols ✚/● : see key b
Number of teratologic chitinozoans per 1000 specimens counted (from: Jaglin & Paris 1992) (Quantitative scale on left hand side axis)
Teratologic specimens of chitinozoans observed during this study
Number of teratologic chitinozoans per 1000 specimens (from: Jaglin & Paris 1992)
interval with teratologic chitinozoans (Jaglin & Paris 1992 + this study)

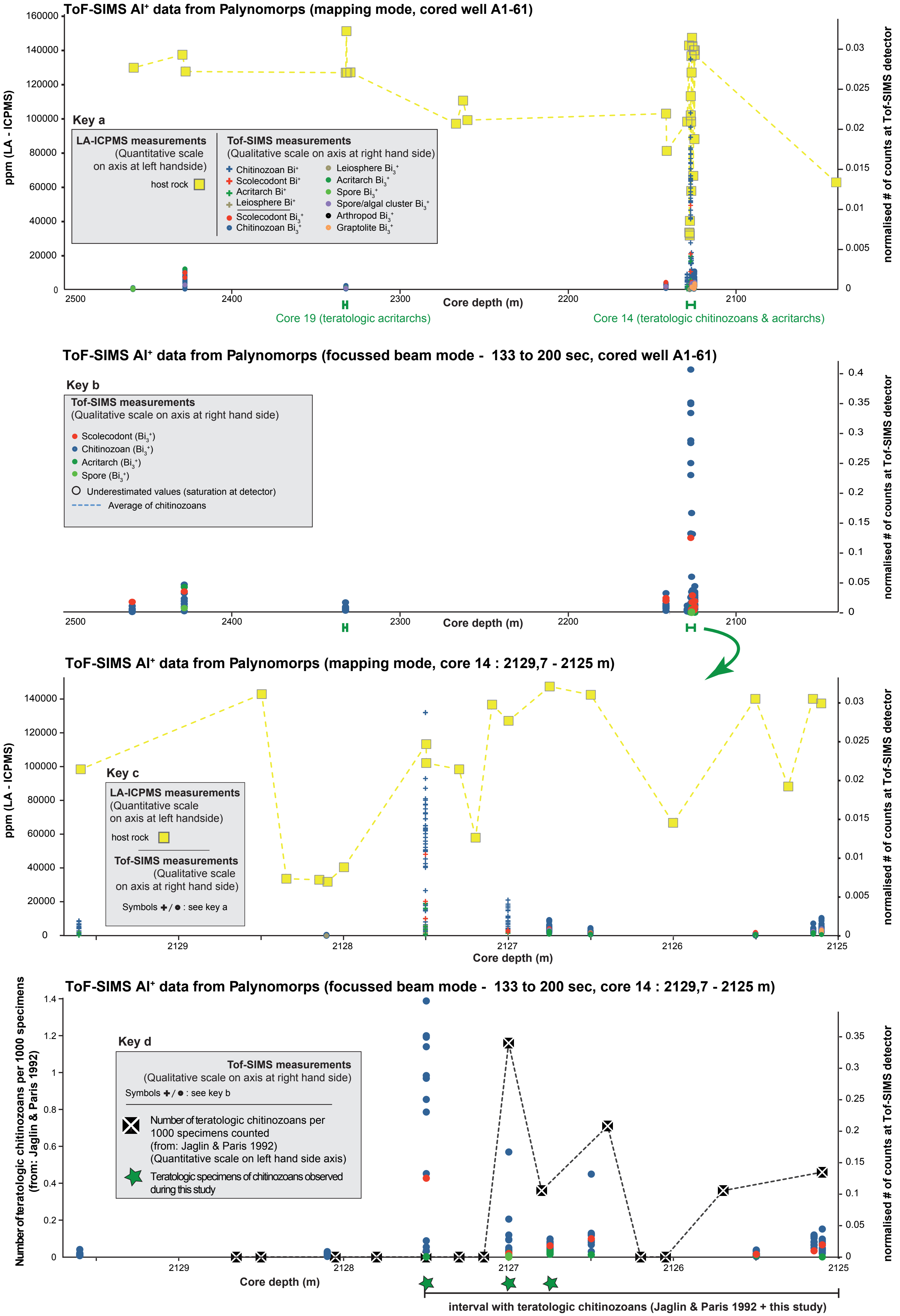
ToF-SIMS Al+ data from Palynomorps (mapping mode, cored well A1-61)
ppm (LA - ICPMS)
normalised # of counts at Tof-SIMS detector
Key a
LA-ICPMS measurements
(Quantitative scale
on axis at left handside)
host rock
Tof-SIMS measurements
(Qualitative scale on axis at right hand side)
Chitinozoan Bi+
Scolecodont Bi+
Acritarch Bi+
Leiosphere Bi+
Scolecodont Bi3+
Chitinozoan Bi3+
Leiosphere Bi3+
Acritarch Bi3+
Spore Bi3+
Spore/algal cluster Bi3+
Arthropod Bi3+
Graptolite Bi3+
Core depth (m)
Core 19 (teratologic acritarchs)
Core 14 (teratologic chitinozoans & acritarchs)
ToF-SIMS Al+ data from Palynomorps (focussed beam mode - 133 to 200 sec, cored well A1-61)
Key b
Tof-SIMS measurements
(Qualitative scale on axis at right hand side)
Scolecodont (Bi3+)
Chitinozoan (Bi3+)
Acritarch (Bi3+)
Spore (Bi3+)
Underestimated values (saturation at detector)
Average of chitinozoans
Core depth (m)
ToF-SIMS Al+ data from Palynomorps (mapping mode, core 14 : 2129,7 - 2125 m)
ppm (LA - ICPMS)
normalised # of counts at Tof-SIMS detector
Key c
LA-ICPMS measurements
(Quantitative scale
on axis at left handside)
host rock
Tof-SIMS measurements
(Qualitative scale
on axis at right hand side)
Symbols ✚/●: see key a
Core depth (m)
ToF-SIMS Al+ data from Palynomorps (focussed beam mode - 133 to 200 sec, core 14 : 2129,7 - 2125 m)
Number of teratologic chitinozoans per 1000 specimens (from: Jaglin & Paris 1992)
normalised # of counts at Tof-SIMS detector
Key d
Tof-SIMS measurements
(Qualitative scale on axis at right hand side)
Symbols ✚/●: see key b
Number of teratologic chitinozoans per 1000 specimens counted
(from: Jaglin & Paris 1992)
(Quantitative scale on left hand side axis)
Teratologic specimens of chitinozoans observed during this study
Core depth (m)
interval with teratologic chitinozoans (Jaglin & Paris 1992 + this study)

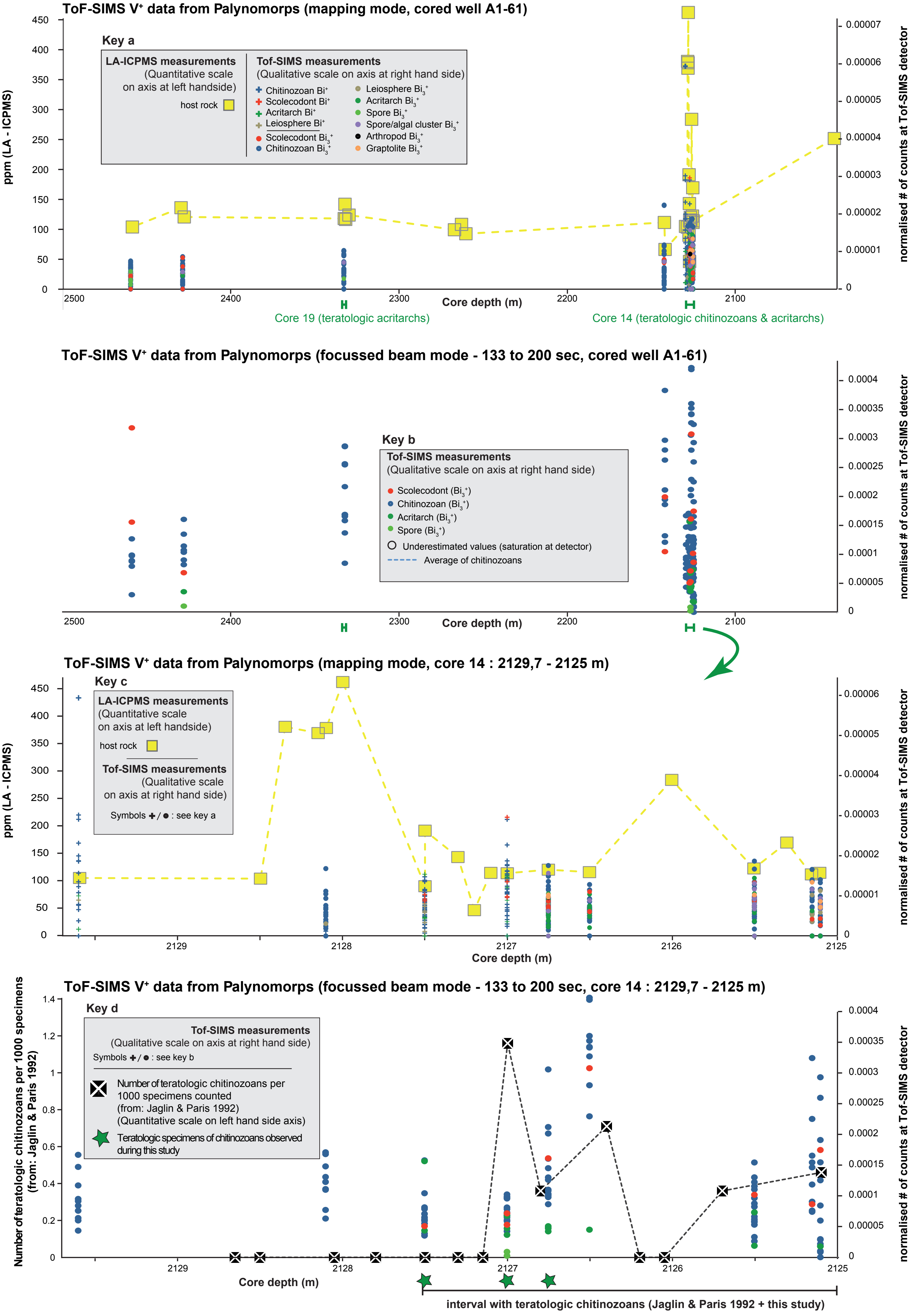
ToF-SIMS V+ data from Palynomorps (mapping mode, cored well A1-61)
Key a
LA-ICPMS measurements
(Quantitative scale on axis at left handside)
host rock
Tof-SIMS measurements
(Qualitative scale on axis at right hand side)
Chitinozoan Bi+
Scolecodont Bi+
Acritarch Bi+
Leiosphere Bi+
Scolecodont Bi3+
Chitinozoan Bi3+
Leiosphere Bi3+
Acritarch Bi3+
Spore Bi3+
Spore/algal cluster Bi3+
Arthropod Bi3+
Graptolite Bi3+
ppm (LA - ICPMS)
normalised # of counts at Tof-SIMS detector
Core depth (m)
Core 19 (teratologic acritarchs)
Core 14 (teratologic chitinozoans & acritarchs)
ToF-SIMS V+ data from Palynomorps (focussed beam mode - 133 to 200 sec, cored well A1-61)
Key b
Tof-SIMS measurements
(Qualitative scale on axis at right hand side)
Scolecodont (Bi3+)
Chitinozoan (Bi3+)
Acritarch (Bi3+)
Spore (Bi3+)
Underestimated values (saturation at detector)
Average of chitinozoans
ToF-SIMS V+ data from Palynomorps (mapping mode, core 14 : 2129,7 - 2125 m)
Key c
LA-ICPMS measurements
(Quantitative scale on axis at left handside)
host rock
Tof-SIMS measurements
(Qualitative scale on axis at right hand side)
Symbols + / ● : see key a
ToF-SIMS V+ data from Palynomorps (focussed beam mode - 133 to 200 sec, core 14 : 2129,7 - 2125 m)
Key d
Tof-SIMS measurements
(Qualitative scale on axis at right hand side)
Symbols + / ● : see key b
Number of teratologic chitinozoans per 1000 specimens counted
(from: Jaglin & Paris 1992)
(Quantitative scale on left hand side axis)
Teratologic specimens of chitinozoans observed during this study
Number of teratologic chitinozoans per 1000 specimens
(from: Jaglin & Paris 1992)
interval with teratologic chitinozoans (Jaglin & Paris 1992 + this study)

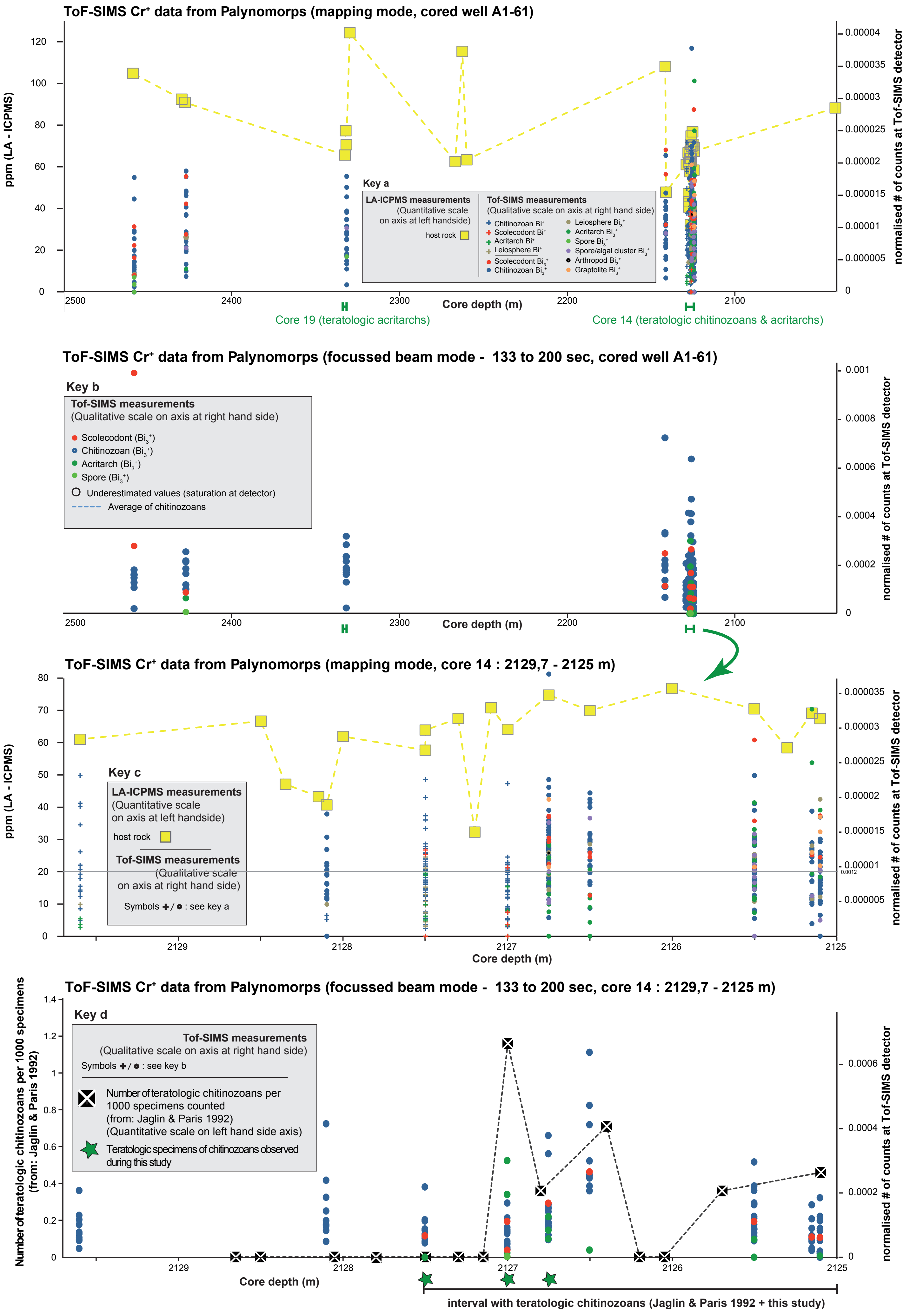
ToF-SIMS Cr+ data from Palynomorps (mapping mode, cored well A1-61)
ppm (LA - ICPMS)
normalised # of counts at Tof-SIMS detector
Key a
LA-ICPMS measurements
(Quantitative scale on axis at left handside)
host rock
Tof-SIMS measurements
(Qualitative scale on axis at right hand side)
Chitinozoan Bi+
Scolecodont Bi+
Acritarch Bi+
Leiosphere Bi+
Scolecodont Bi3+
Chitinozoan Bi3+
Leiosphere Bi3+
Acritarch Bi3+
Spore Bi3+
Spore/algal cluster Bi3+
Arthropod Bi3+
Graptolite Bi3+
Core depth (m)
Core 19 (teratologic acritarchs)
Core 14 (teratologic chitinozoans & acritarchs)
ToF-SIMS Cr+ data from Palynomorps (focussed beam mode - 133 to 200 sec, cored well A1-61)
Key b
Tof-SIMS measurements
(Qualitative scale on axis at right hand side)
Scolecodont (Bi3+)
Chitinozoan (Bi3+)
Acritarch (Bi3+)
Spore (Bi3+)
Underestimated values (saturation at detector)
Average of chitinozoans
normalised # of counts at Tof-SIMS detector
Core depth (m)
ToF-SIMS Cr+ data from Palynomorps (mapping mode, core 14 : 2129,7 - 2125 m)
ppm (LA - ICPMS)
Key c
LA-ICPMS measurements
(Quantitative scale on axis at left handside)
host rock
Tof-SIMS measurements
(Qualitative scale on axis at right hand side)
Symbols ✚/●: see key a
normalised # of counts at Tof-SIMS detector
Core depth (m)
ToF-SIMS Cr+ data from Palynomorps (focussed beam mode - 133 to 200 sec, core 14 : 2129,7 - 2125 m)
Number of teratologic chitinozoans per 1000 specimens (from: Jaglin & Paris 1992)
Key d
Tof-SIMS measurements
(Qualitative scale on axis at right hand side)
Symbols ✚/●: see key b
Number of teratologic chitinozoans per 1000 specimens counted (from: Jaglin & Paris 1992) (Quantitative scale on left hand side axis)
Teratologic specimens of chitinozoans observed during this study
normalised # of counts at Tof-SIMS detector
Core depth (m)
interval with teratologic chitinozoans (Jaglin & Paris 1992 + this study)

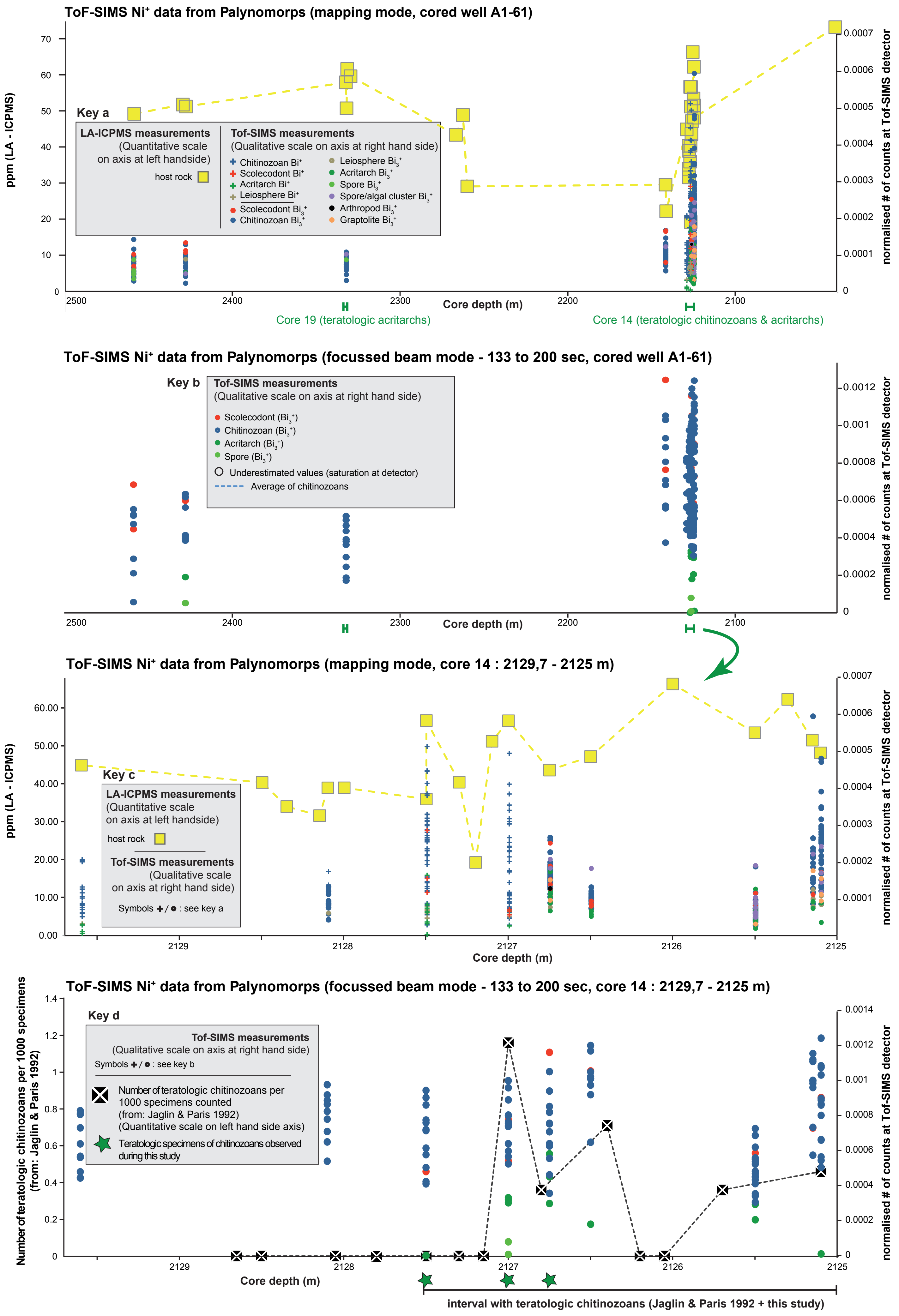

ToF-SIMS Ni+ data from Palynomorps (mapping mode, cored well A1-61)
ppm (LA - ICPMS)
normalised # of counts at Tof-SIMS detector
Key a
LA-ICPMS measurements
(Quantitative scale on axis at left handside)
host rock
Tof-SIMS measurements
(Qualitative scale on axis at right hand side)
Chitinozoan Bi+
Scolecodont Bi+
Acritarch Bi+
Leiosphere Bi+
Scolecodont Bi3+
Chitinozoan Bi3+
Leiosphere Bi3+
Acritarch Bi3+
Spore Bi3+
Spore/algal cluster Bi3+
Arthropod Bi3+
Graptolite Bi3+
Core depth (m)
Core 19 (teratologic acritarchs)
Core 14 (teratologic chitinozoans & acritarchs)
ToF-SIMS Ni+ data from Palynomorps (focussed beam mode - 133 to 200 sec, cored well A1-61)
Key b
Tof-SIMS measurements
(Qualitative scale on axis at right hand side)
Scolecodont (Bi3+)
Chitinozoan (Bi3+)
Acritarch (Bi3+)
Spore (Bi3+)
Underestimated values (saturation at detector)
Average of chitinozoans
Core depth (m)
ToF-SIMS Ni+ data from Palynomorps (mapping mode, core 14 : 2129,7 - 2125 m)
Key c
LA-ICPMS measurements
(Quantitative scale on axis at left handside)
host rock
Tof-SIMS measurements
(Qualitative scale on axis at right hand side)
Symbols ✚ / ● : see key a
Core depth (m)
ToF-SIMS Ni+ data from Palynomorps (focussed beam mode - 133 to 200 sec, core 14 : 2129,7 - 2125 m)
Number of teratologic chitinozoans per 1000 specimens (from: Jaglin & Paris 1992)
Key d
Tof-SIMS measurements
(Qualitative scale on axis at right hand side)
Symbols ✚ / ● : see key b
Number of teratologic chitinozoans per 1000 specimens counted
(from: Jaglin & Paris 1992)
(Quantitative scale on left hand side axis)
Teratologic specimens of chitinozoans observed during this study
Core depth (m)
interval with teratologic chitinozoans (Jaglin & Paris 1992 + this study)

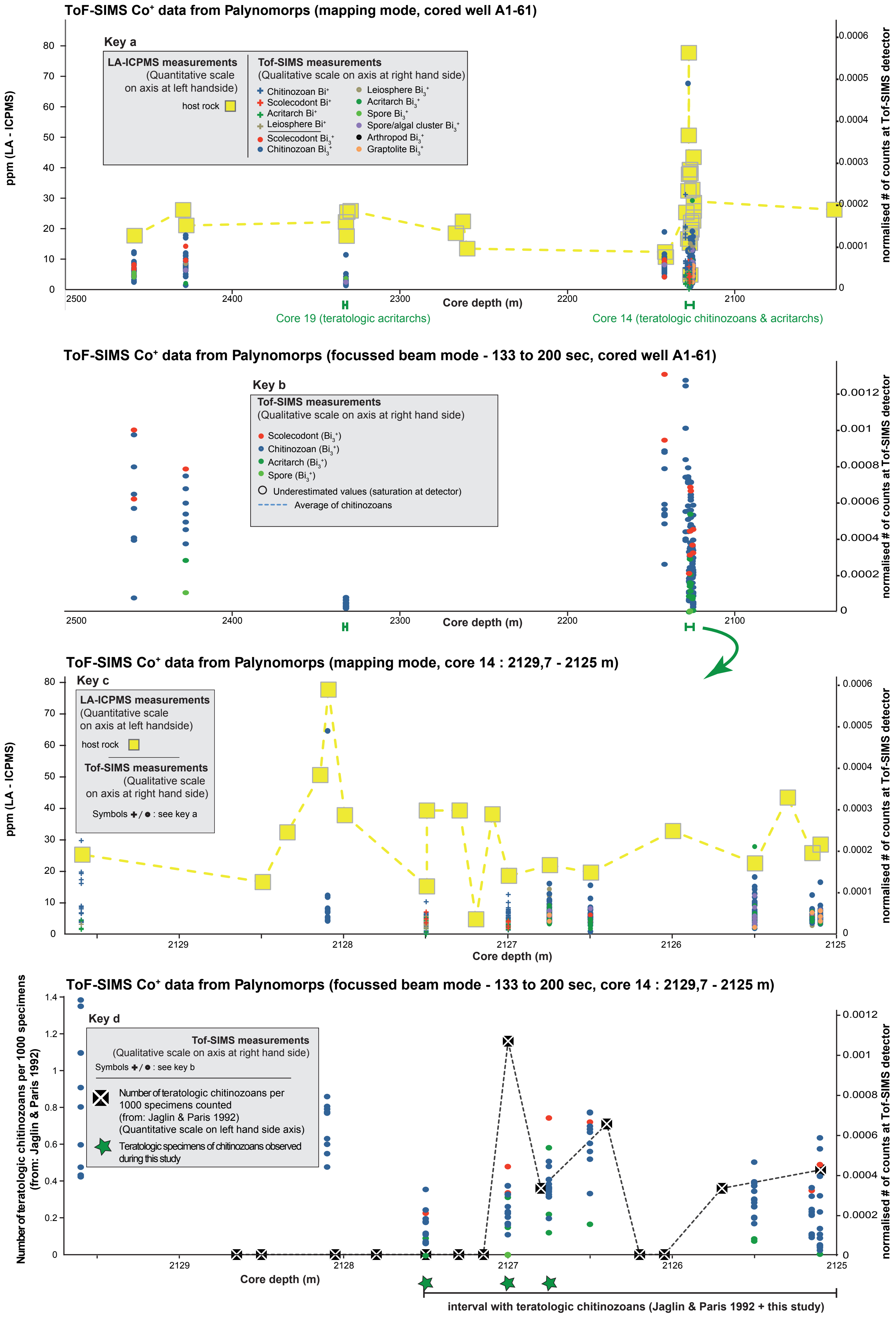

ToF-SIMS Co+ data from Palynomorps (mapping mode, cored well A1-61)
Key a
LA-ICPMS measurements
(Quantitative scale on axis at left handside)
host rock
Tof-SIMS measurements
(Qualitative scale on axis at right hand side)
Chitinozoan Bi+
Scolecodont Bi+
Acritarch Bi+
Leiosphere Bi+
Scolecodont Bi3+
Chitinozoan Bi3+
Leiosphere Bi3+
Acritarch Bi3+
Spore Bi3+
Spore/algal cluster Bi3+
Arthropod Bi3+
Graptolite Bi3+
ppm (LA - ICPMS)
normalised # of counts at Tof-SIMS detector
Core depth (m)
Core 19 (teratologic acritarchs)
Core 14 (teratologic chitinozoans & acritarchs)
ToF-SIMS Co+ data from Palynomorps (focussed beam mode - 133 to 200 sec, cored well A1-61)
Key b
Tof-SIMS measurements
(Qualitative scale on axis at right hand side)
Scolecodont (Bi3+)
Chitinozoan (Bi3+)
Acritarch (Bi3+)
Spore (Bi3+)
Underestimated values (saturation at detector)
Average of chitinozoans
normalised # of counts at Tof-SIMS detector
Core depth (m)
ToF-SIMS Co+ data from Palynomorps (mapping mode, core 14 : 2129,7 - 2125 m)
Key c
LA-ICPMS measurements
(Quantitative scale on axis at left handside)
host rock
Tof-SIMS measurements
(Qualitative scale on axis at right hand side)
Symbols ✚ / ● : see key a
ppm (LA - ICPMS)
normalised # of counts at Tof-SIMS detector
Core depth (m)
ToF-SIMS Co+ data from Palynomorps (focussed beam mode - 133 to 200 sec, core 14 : 2129,7 - 2125 m)
Key d
Tof-SIMS measurements
(Qualitative scale on axis at right hand side)
Symbols ✚ / ● : see key b
Number of teratologic chitinozoans per 1000 specimens counted (from: Jaglin & Paris 1992) (Quantitative scale on left hand side axis)
Teratologic specimens of chitinozoans observed during this study
Number of teratologic chitinozoans per 1000 specimens (from: Jaglin & Paris 1992)
normalised # of counts at Tof-SIMS detector
Core depth (m)
interval with teratologic chitinozoans (Jaglin & Paris 1992 + this study)

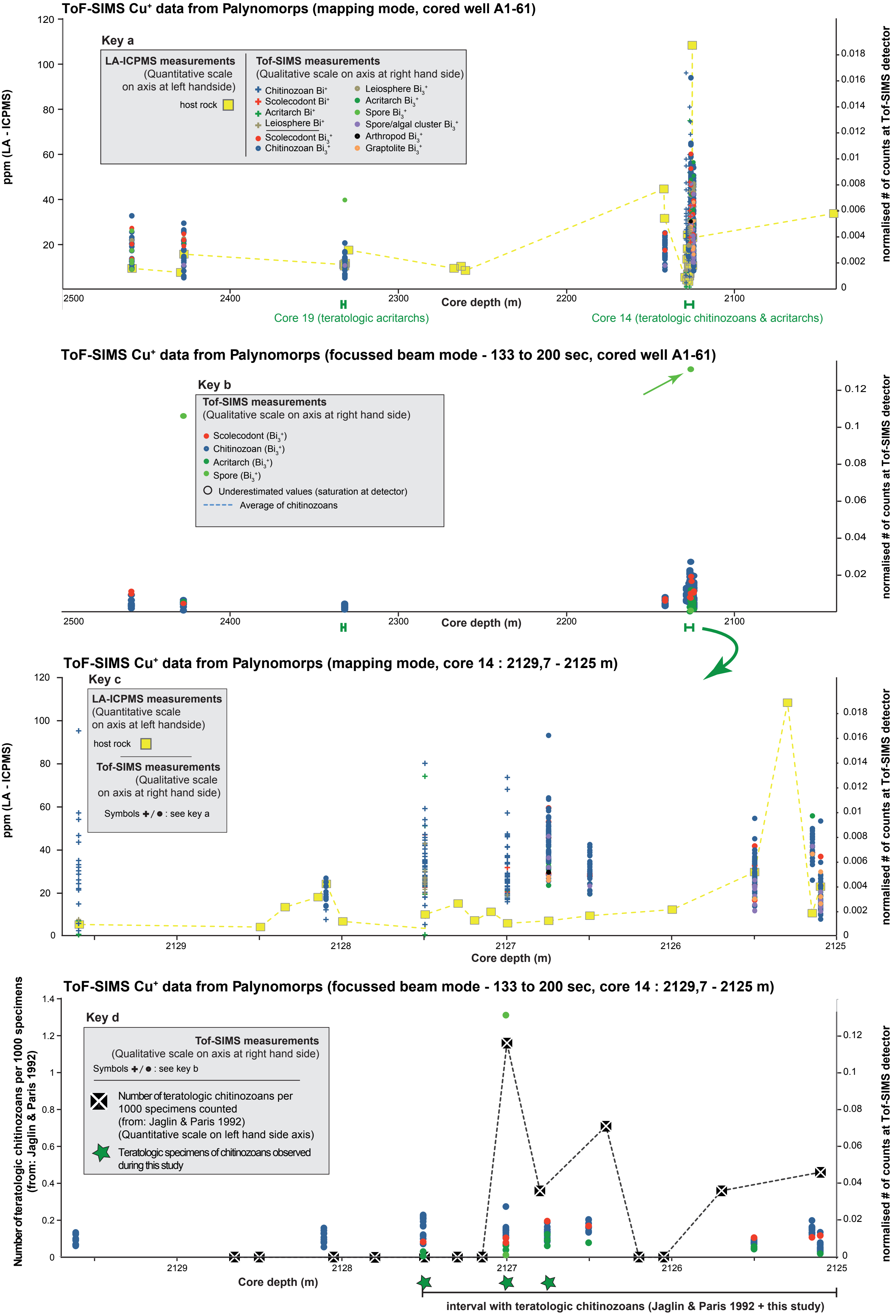

ToF-SIMS Cu+ data from Palynomorps (mapping mode, cored well A1-61)
Key a
LA-ICPMS measurements
(Quantitative scale on axis at left handside)
host rock
Tof-SIMS measurements
(Qualitative scale on axis at right hand side)
Chitinozoan Bi+
Scolecodont Bi+
Acritarch Bi+
Leiosphere Bi+
Scolecodont Bi3+
Chitinozoan Bi3+
Leiosphere Bi3+
Acritarch Bi3+
Spore Bi3+
Spore/algal cluster Bi3+
Arthropod Bi3+
Graptolite Bi3+
ppm (LA - ICPMS)
normalised # of counts at Tof-SIMS detector
Core depth (m)
Core 19 (teratologic acritarchs)
Core 14 (teratologic chitinozoans & acritarchs)
ToF-SIMS Cu+ data from Palynomorps (focussed beam mode - 133 to 200 sec, cored well A1-61)
Key b
Tof-SIMS measurements
(Qualitative scale on axis at right hand side)
Scolecodont (Bi3+)
Chitinozoan (Bi3+)
Acritarch (Bi3+)
Spore (Bi3+)
Underestimated values (saturation at detector)
Average of chitinozoans
ToF-SIMS Cu+ data from Palynomorps (mapping mode, core 14 : 2129,7 - 2125 m)
Key c
LA-ICPMS measurements
(Quantitative scale on axis at left handside)
host rock
Tof-SIMS measurements
(Qualitative scale on axis at right hand side)
Symbols +/●: see key a
ToF-SIMS Cu+ data from Palynomorps (focussed beam mode - 133 to 200 sec, core 14 : 2129,7 - 2125 m)
Key d
Tof-SIMS measurements
(Qualitative scale on axis at right hand side)
Symbols +/●: see key b
Number of teratologic chitinozoans per 1000 specimens counted
(from: Jaglin & Paris 1992)
(Quantitative scale on left hand side axis)
Teratologic specimens of chitinozoans observed during this study
Number of teratologic chitinozoans per 1000 specimens (from: Jaglin & Paris 1992)
interval with teratologic chitinozoans (Jaglin & Paris 1992 + this study)

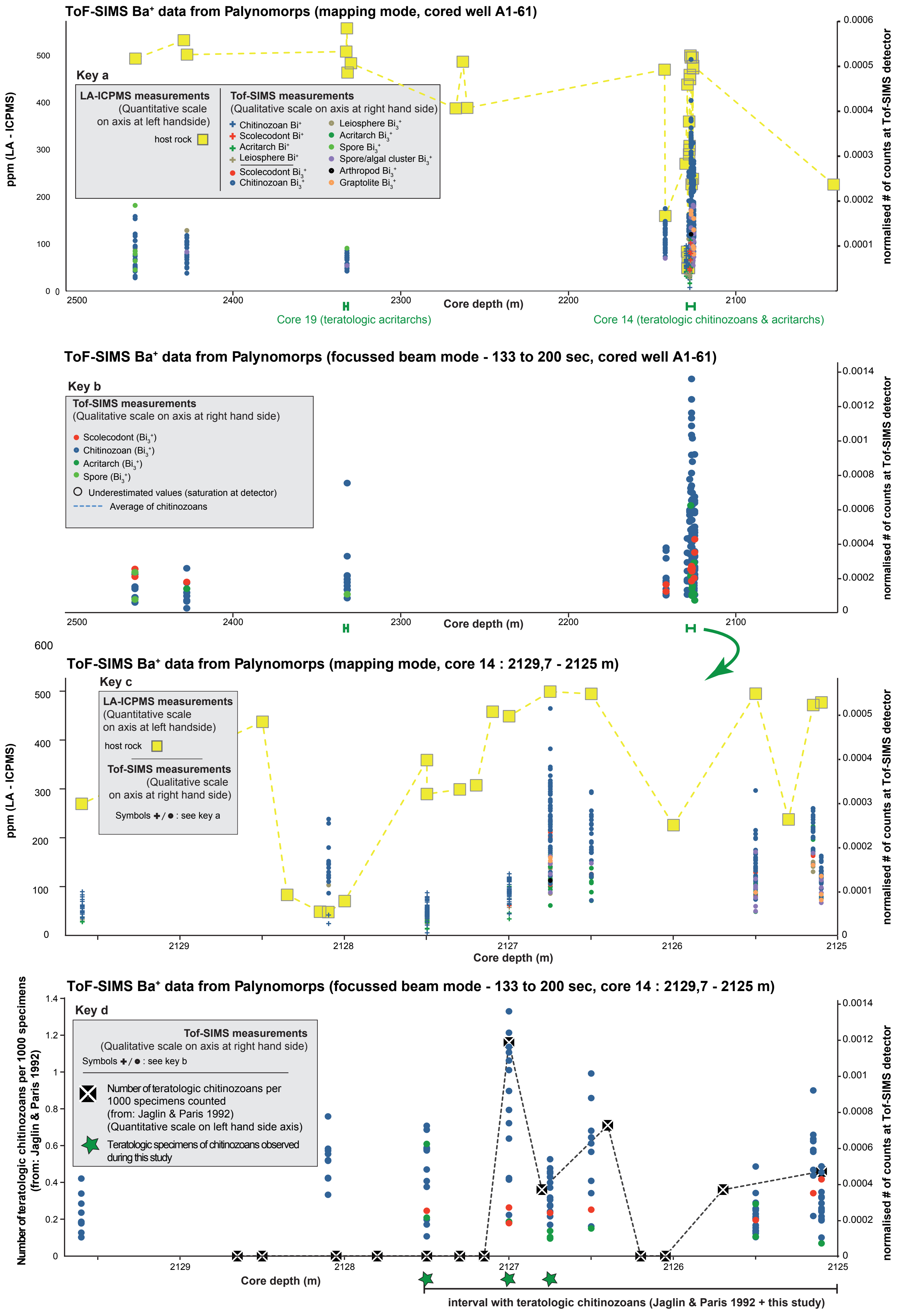
ToF-SIMS Ba+ data from Palynomorps (mapping mode, cored well A1-61)
Key a
LA-ICPMS measurements
(Quantitative scale on axis at left handside)
host rock
Tof-SIMS measurements
(Qualitative scale on axis at right hand side)
Chitinozoan Bi+
Scolecodont Bi+
Acritarch Bi+
Leiosphere Bi+
Scolecodont Bi3+
Chitinozoan Bi3+
Leiosphere Bi3+
Acritarch Bi3+
Spore Bi3+
Spore/algal cluster Bi3+
Arthropod Bi3+
Graptolite Bi3+
ppm (LA - ICPMS)
normalised # of counts at Tof-SIMS detector
Core depth (m)
Core 19 (teratologic acritarchs)
Core 14 (teratologic chitinozoans & acritarchs)
ToF-SIMS Ba+ data from Palynomorps (focussed beam mode - 133 to 200 sec, cored well A1-61)
Key b
Tof-SIMS measurements
(Qualitative scale on axis at right hand side)
Scolecodont (Bi3+)
Chitinozoan (Bi3+)
Acritarch (Bi3+)
Spore (Bi3+)
Underestimated values (saturation at detector)
Average of chitinozoans
ToF-SIMS Ba+ data from Palynomorps (mapping mode, core 14 : 2129,7 - 2125 m)
Key c
LA-ICPMS measurements
(Quantitative scale on axis at left handside)
host rock
Tof-SIMS measurements
(Qualitative scale on axis at right hand side)
Symbols ✚/●: see key a
ToF-SIMS Ba+ data from Palynomorps (focussed beam mode - 133 to 200 sec, core 14 : 2129,7 - 2125 m)
Key d
Tof-SIMS measurements
(Qualitative scale on axis at right hand side)
Symbols ✚/●: see key b
Number of teratologic chitinozoans per 1000 specimens counted
(from: Jaglin & Paris 1992)
(Quantitative scale on left hand side axis)
Teratologic specimens of chitinozoans observed during this study
Number of teratologic chitinozoans per 1000 specimens
(from: Jaglin & Paris 1992)
interval with teratologic chitinozoans (Jaglin & Paris 1992 + this study)

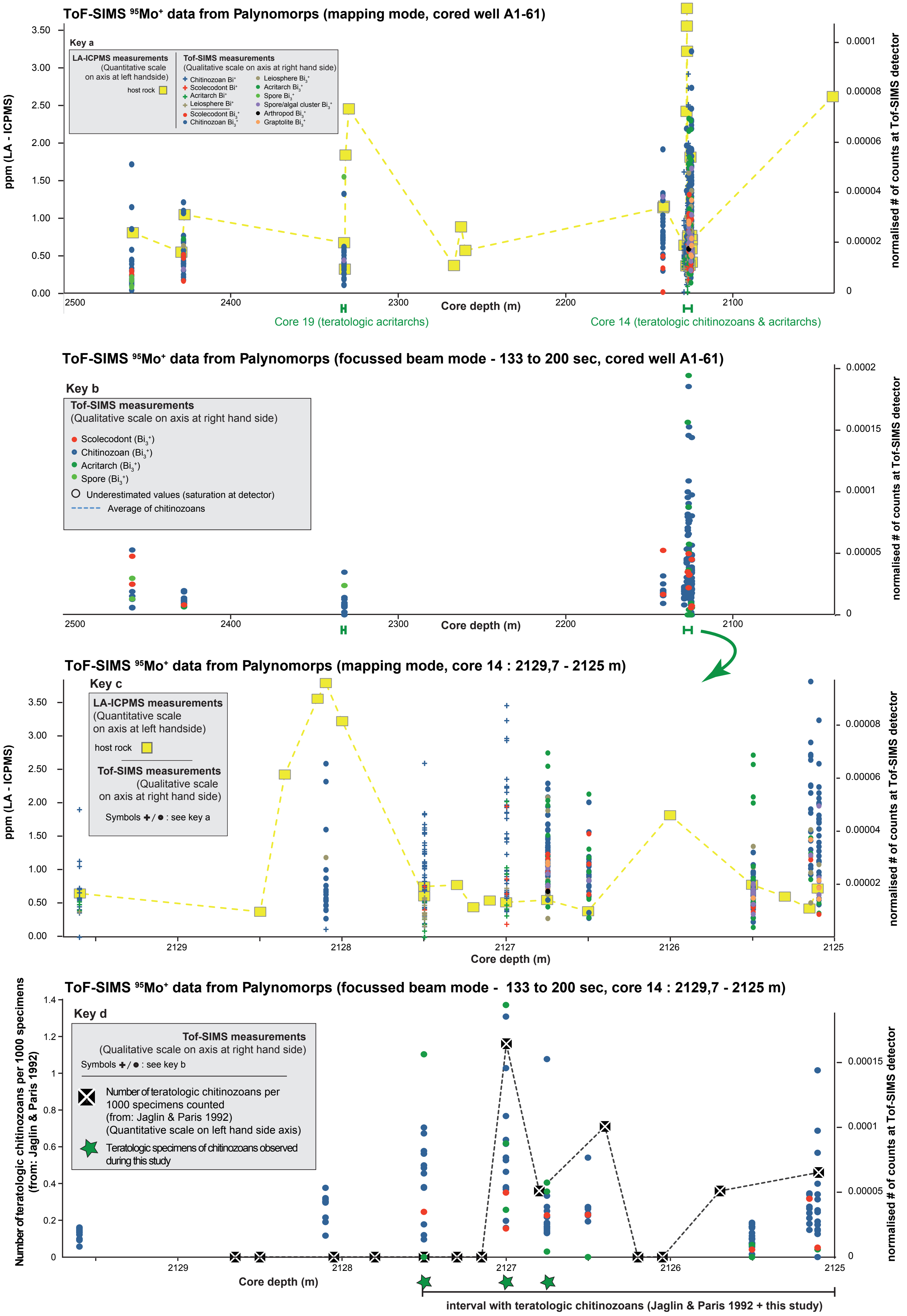
ToF-SIMS ⁹⁵Mo⁺ data from Palynomorps (mapping mode, cored well A1-61)
Key a
LA-ICPMS measurements
(Quantitative scale on axis at left handside)
host rock
Tof-SIMS measurements
(Qualitative scale on axis at right hand side)
Chitinozoan Bi⁺
Scolecodont Bi⁺
Acritarch Bi⁺
Leiosphere Bi⁺
Scolecodont Bi₃⁺
Chitinozoan Bi₃⁺
Leiosphere Bi₃⁺
Acritarch Bi₃⁺
Spore Bi₃⁺
Spore/algal cluster Bi₃⁺
Arthropod Bi₃⁺
Graptolite Bi₃⁺
ppm (LA - ICPMS)
normalised # of counts at Tof-SIMS detector
Core depth (m)
Core 19 (teratologic acritarchs)
Core 14 (teratologic chitinozoans & acritarchs)
ToF-SIMS ⁹⁵Mo⁺ data from Palynomorps (focussed beam mode - 133 to 200 sec, cored well A1-61)
Key b
Tof-SIMS measurements
(Qualitative scale on axis at right hand side)
Scolecodont (Bi₃⁺)
Chitinozoan (Bi₃⁺)
Acritarch (Bi₃⁺)
Spore (Bi₃⁺)
Underestimated values (saturation at detector)
Average of chitinozoans
normalised # of counts at Tof-SIMS detector
Core depth (m)
ToF-SIMS ⁹⁵Mo⁺ data from Palynomorps (mapping mode, core 14 : 2129,7 - 2125 m)
Key c
LA-ICPMS measurements
(Quantitative scale on axis at left handside)
host rock
Tof-SIMS measurements
(Qualitative scale on axis at right hand side)
Symbols ✚/● : see key a
ppm (LA - ICPMS)
normalised # of counts at Tof-SIMS detector
Core depth (m)
ToF-SIMS ⁹⁵Mo⁺ data from Palynomorps (focussed beam mode - 133 to 200 sec, core 14 : 2129,7 - 2125 m)
Key d
Tof-SIMS measurements
(Qualitative scale on axis at right hand side)
Symbols ✚/● : see key b
Number of teratologic chitinozoans per 1000 specimens counted
(from: Jaglin & Paris 1992)
(Quantitative scale on left hand side axis)
Teratologic specimens of chitinozoans observed during this study
Number of teratologic chitinozoans per 1000 specimens (from: Jaglin & Paris 1992)
normalised # of counts at Tof-SIMS detector
Core depth (m)
interval with teratologic chitinozoans (Jaglin & Paris 1992 + this study)

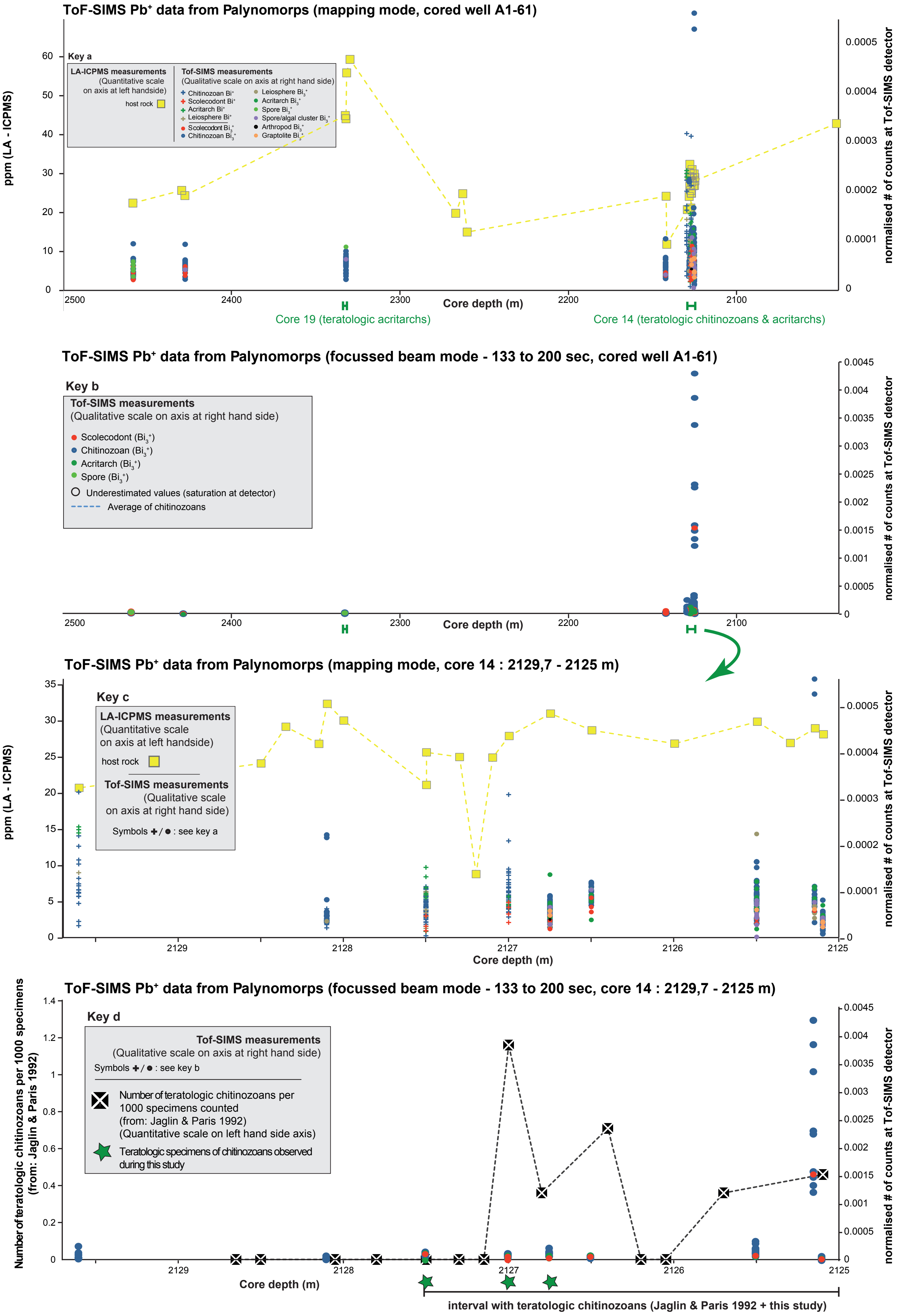
ToF-SIMS Pb+ data from Palynomorps (mapping mode, cored well A1-61)
ppm (LA - ICPMS)
normalised # of counts at Tof-SIMS detector
Key a
LA-ICPMS measurements
(Quantitative scale on axis at left handside)
host rock
Tof-SIMS measurements
(Qualitative scale on axis at right hand side)
Chitinozoan Bi+
Scolecodont Bi+
Acritarch Bi+
Leiosphere Bi+
Scolecodont Bi3+
Chitinozoan Bi3+
Leiosphere Bi3+
Acritarch Bi3+
Spore Bi3+
Spore/algal cluster Bi3+
Arthropod Bi3+
Graptolite Bi3+
0
10
20
30
40
50
60
0.0001
0.0002
0.0003
0.0004
0.0005
2500
2400
2300
2200
2100
Core depth (m)
Core 19 (teratologic acritarchs)
Core 14 (teratologic chitinozoans & acritarchs)
ToF-SIMS Pb+ data from Palynomorps (focussed beam mode - 133 to 200 sec, cored well A1-61)
Key b
Tof-SIMS measurements
(Qualitative scale on axis at right hand side)
Scolecodont (Bi3+)
Chitinozoan (Bi3+)
Acritarch (Bi3+)
Spore (Bi3+)
Underestimated values (saturation at detector)
Average of chitinozoans
0.0005
0.001
0.0015
0.002
0.0025
0.003
0.0035
0.004
0.0045
ToF-SIMS Pb+ data from Palynomorps (mapping mode, core 14 : 2129,7 - 2125 m)
Key c
LA-ICPMS measurements
(Quantitative scale on axis at left handside)
host rock
Tof-SIMS measurements
(Qualitative scale on axis at right hand side)
Symbols ✚/●: see key a
5
15
25
35
2129
2128
2127
2126
2125
ToF-SIMS Pb+ data from Palynomorps (focussed beam mode - 133 to 200 sec, core 14 : 2129,7 - 2125 m)
Number of teratologic chitinozoans per 1000 specimens (from: Jaglin & Paris 1992)
Key d
Tof-SIMS measurements
(Qualitative scale on axis at right hand side)
Symbols ✚/●: see key b
Number of teratologic chitinozoans per 1000 specimens counted
(from: Jaglin & Paris 1992)
(Quantitative scale on left hand side axis)
Teratologic specimens of chitinozoans observed during this study
0.2
0.4
0.6
0.8
1
1.2
1.4
interval with teratologic chitinozoans (Jaglin & Paris 1992 + this study)

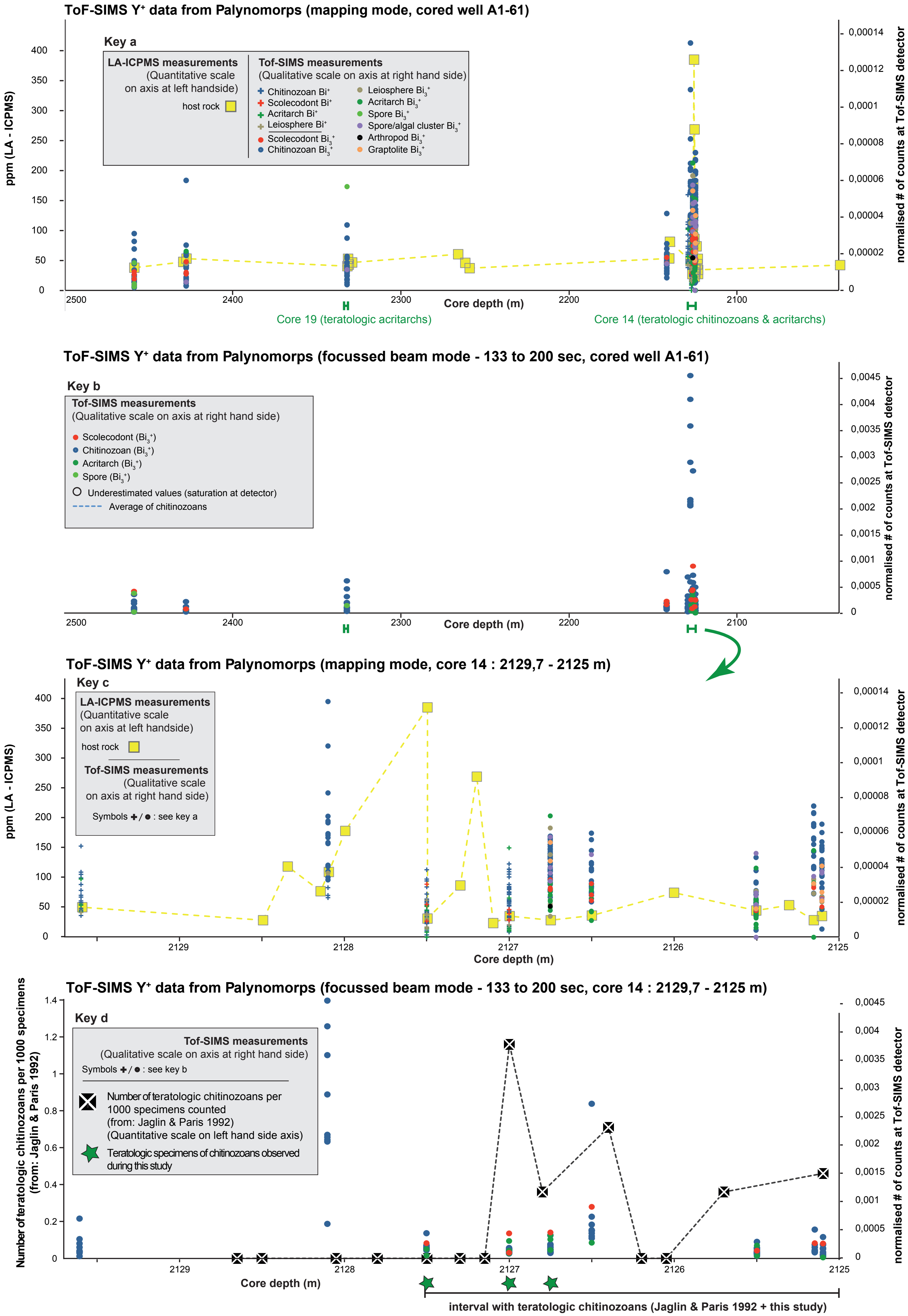
ToF-SIMS Y+ data from Palynomorps (mapping mode, cored well A1-61)
Key a
LA-ICPMS measurements
(Quantitative scale on axis at left handside)
host rock
Tof-SIMS measurements
(Qualitative scale on axis at right hand side)
Chitinozoan Bi+
Scolecodont Bi+
Acritarch Bi+
Leiosphere Bi+
Scolecodont Bi3+
Chitinozoan Bi3+
Leiosphere Bi3+
Acritarch Bi3+
Spore Bi3+
Spore/algal cluster Bi3+
Arthropod Bi3+
Graptolite Bi3+
ppm (LA - ICPMS)
normalised # of counts at Tof-SIMS detector
Core depth (m)
Core 19 (teratologic acritarchs)
Core 14 (teratologic chitinozoans & acritarchs)
ToF-SIMS Y+ data from Palynomorps (focussed beam mode - 133 to 200 sec, cored well A1-61)
Key b
Tof-SIMS measurements
(Qualitative scale on axis at right hand side)
Scolecodont (Bi3+)
Chitinozoan (Bi3+)
Acritarch (Bi3+)
Spore (Bi3+)
Underestimated values (saturation at detector)
Average of chitinozoans
normalised # of counts at Tof-SIMS detector
Core depth (m)
ToF-SIMS Y+ data from Palynomorps (mapping mode, core 14 : 2129,7 - 2125 m)
Key c
LA-ICPMS measurements
(Quantitative scale on axis at left handside)
host rock
Tof-SIMS measurements
(Qualitative scale on axis at right hand side)
Symbols ✚ / ● : see key a
ppm (LA - ICPMS)
normalised # of counts at Tof-SIMS detector
Core depth (m)
ToF-SIMS Y+ data from Palynomorps (focussed beam mode - 133 to 200 sec, core 14 : 2129,7 - 2125 m)
Key d
Tof-SIMS measurements
(Qualitative scale on axis at right hand side)
Symbols ✚ / ● : see key b
Number of teratologic chitinozoans per 1000 specimens counted
(from: Jaglin & Paris 1992)
(Quantitative scale on left hand side axis)
Teratologic specimens of chitinozoans observed during this study
Number of teratologic chitinozoans per 1000 specimens (from: Jaglin & Paris 1992)
normalised # of counts at Tof-SIMS detector
Core depth (m)
interval with teratologic chitinozoans (Jaglin & Paris 1992 + this study)

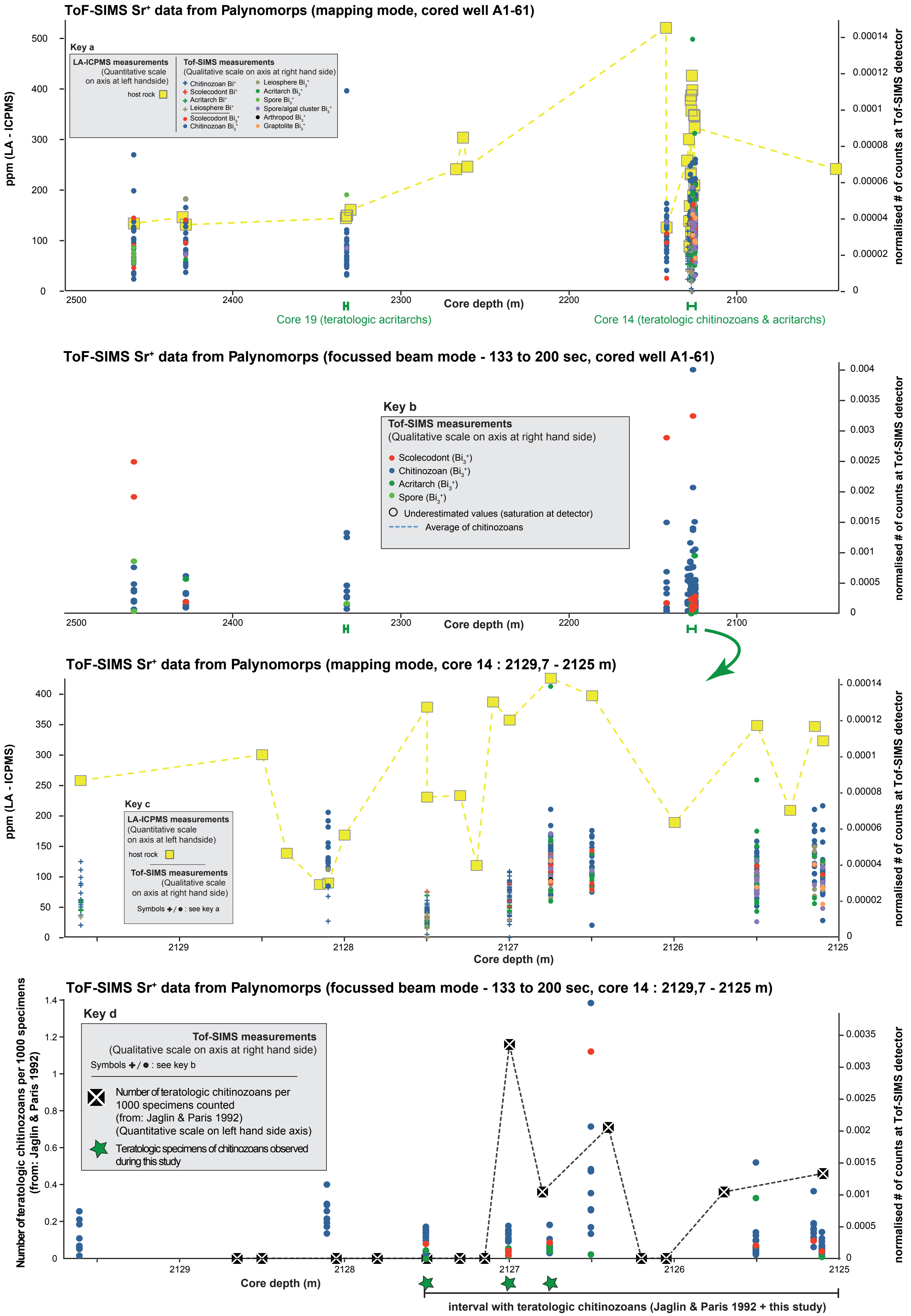
ToF-SIMS Sr+ data from Palynomorps (mapping mode, cored well A1-61)
ppm (LA - ICPMS)
Key a
LA-ICPMS measurements
(Quantitative scale
on axis at left handside)
host rock
Tof-SIMS measurements
(Qualitative scale on axis at right hand side)
Chitinozoan Bi+
Scolecodont Bi+
Acritarch Bi+
Leiosphere Bi+
Scolecodont Bi3+
Chitinozoan Bi3+
Leiosphere Bi3+
Acritarch Bi3+
Spore Bi3+
Spore/algal cluster Bi3+
Arthropod Bi3+
Graptolite Bi3+
Core depth (m)
normalised # of counts at Tof-SIMS detector
Core 19 (teratologic acritarchs)
Core 14 (teratologic chitinozoans & acritarchs)
ToF-SIMS Sr+ data from Palynomorps (focussed beam mode - 133 to 200 sec, cored well A1-61)
Key b
Tof-SIMS measurements
(Qualitative scale on axis at right hand side)
Scolecodont (Bi3+)
Chitinozoan (Bi3+)
Acritarch (Bi3+)
Spore (Bi3+)
Underestimated values (saturation at detector)
Average of chitinozoans
Core depth (m)
normalised # of counts at Tof-SIMS detector
ToF-SIMS Sr+ data from Palynomorps (mapping mode, core 14 : 2129,7 - 2125 m)
ppm (LA - ICPMS)
Key c
LA-ICPMS measurements
(Quantitative scale
on axis at left handside)
host rock
TOF-SIMS measurements
(Qualitative scale
on axis at right hand side)
Symbols ✚/●: see key a
Core depth (m)
normalised # of counts at Tof-SIMS detector
ToF-SIMS Sr+ data from Palynomorps (focussed beam mode - 133 to 200 sec, core 14 : 2129,7 - 2125 m)
Number of teratologic chitinozoans per 1000 specimens (from: Jaglin & Paris 1992)
Key d
Tof-SIMS measurements
(Qualitative scale on axis at right hand side)
Symbols ✚/●: see key b
Number of teratologic chitinozoans per 1000 specimens counted
(from: Jaglin & Paris 1992)
(Quantitative scale on left hand side axis)
Teratologic specimens of chitinozoans observed during this study
Core depth (m)
normalised # of counts at Tof-SIMS detector
interval with teratologic chitinozoans (Jaglin & Paris 1992 + this study)

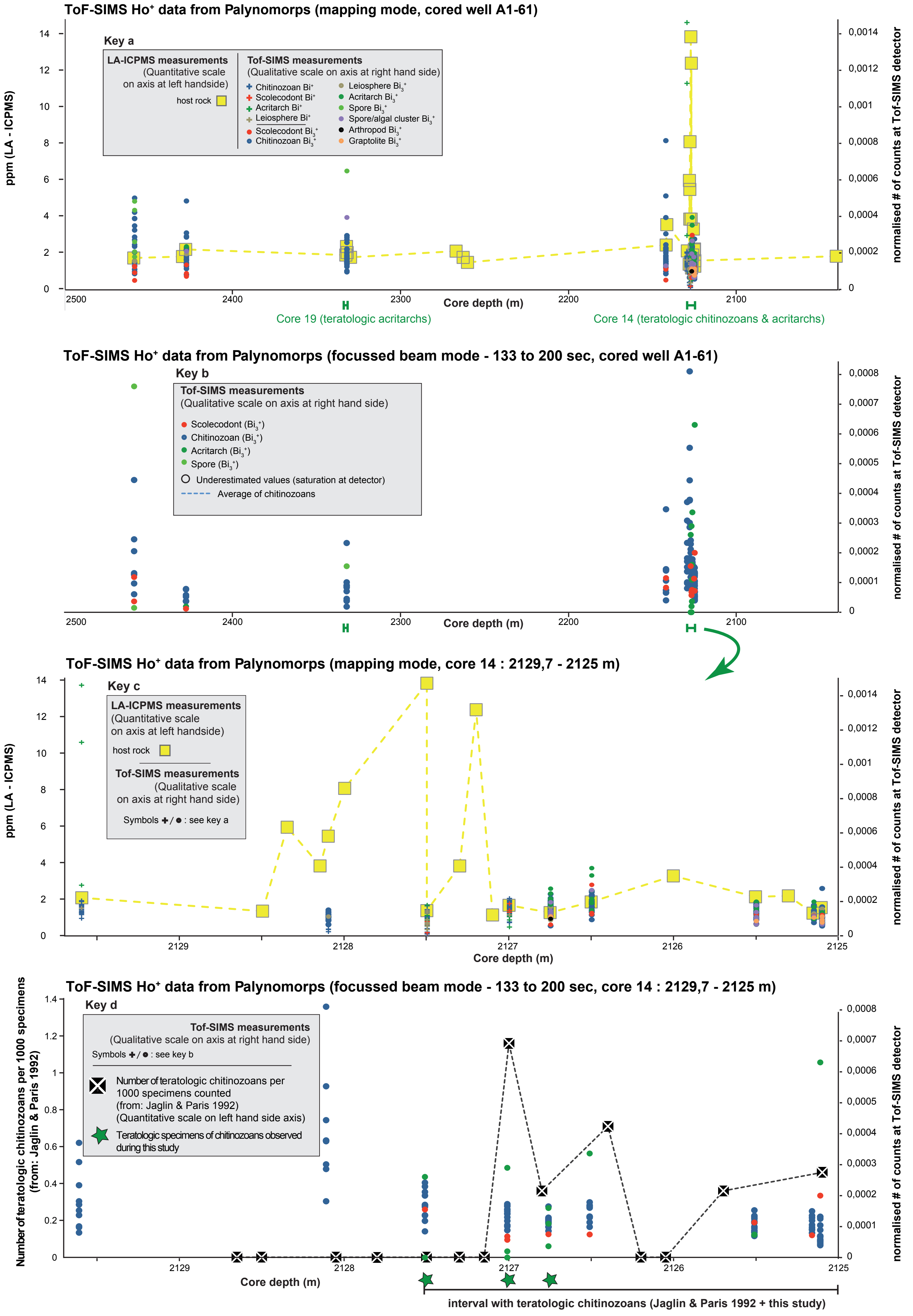

ToF-SIMS Ho+ data from Palynomorps (mapping mode, cored well A1-61)
Key a
LA-ICPMS measurements
(Quantitative scale on axis at left handside)
host rock
Tof-SIMS measurements
(Qualitative scale on axis at right hand side)
Chitinozoan Bi+
Scolecodont Bi+
Acritarch Bi+
Leiosphere Bi+
Scolecodont Bi3+
Chitinozoan Bi3+
Leiosphere Bi3+
Acritarch Bi3+
Spore Bi3+
Spore/algal cluster Bi3+
Arthropod Bi3+
Graptolite Bi3+
ppm (LA - ICPMS)
normalised # of counts at Tof-SIMS detector
Core depth (m)
Core 19 (teratologic acritarchs)
Core 14 (teratologic chitinozoans & acritarchs)
ToF-SIMS Ho+ data from Palynomorps (focussed beam mode - 133 to 200 sec, cored well A1-61)
Key b
Tof-SIMS measurements
(Qualitative scale on axis at right hand side)
Scolecodont (Bi3+)
Chitinozoan (Bi3+)
Acritarch (Bi3+)
Spore (Bi3+)
Underestimated values (saturation at detector)
Average of chitinozoans
ToF-SIMS Ho+ data from Palynomorps (mapping mode, core 14 : 2129,7 - 2125 m)
Key c
LA-ICPMS measurements
(Quantitative scale on axis at left handside)
host rock
Tof-SIMS measurements
(Qualitative scale on axis at right hand side)
Symbols ✚ / ● : see key a
ToF-SIMS Ho+ data from Palynomorps (focussed beam mode - 133 to 200 sec, core 14 : 2129,7 - 2125 m)
Key d
Tof-SIMS measurements
(Qualitative scale on axis at right hand side)
Symbols ✚ / ● : see key b
Number of teratologic chitinozoans per 1000 specimens counted (from: Jaglin & Paris 1992) (Quantitative scale on left hand side axis)
Teratologic specimens of chitinozoans observed during this study
Number of teratologic chitinozoans per 1000 specimens (from: Jaglin & Paris 1992)
interval with teratologic chitinozoans (Jaglin & Paris 1992 + this study)

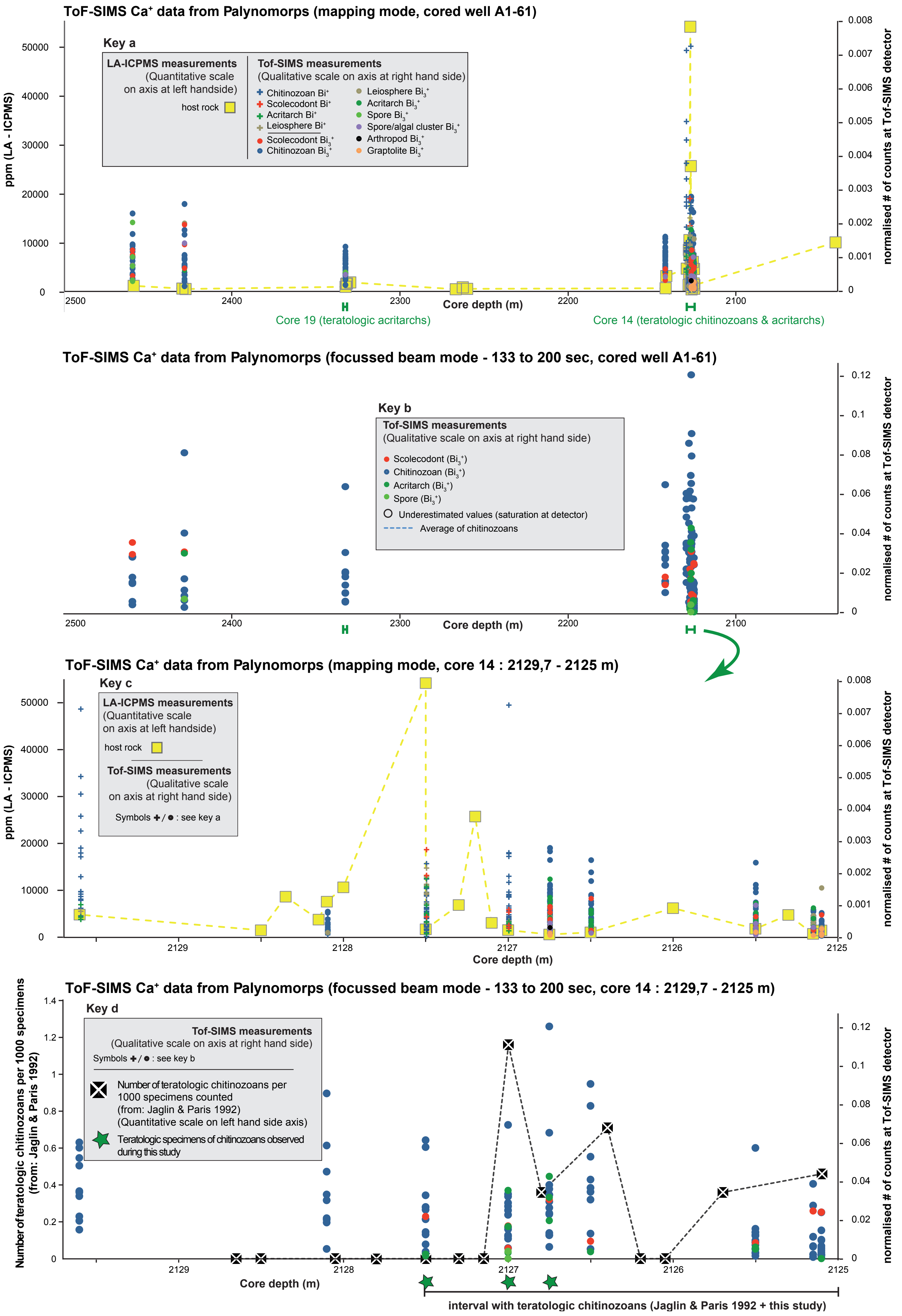

ToF-SIMS Ca+ data from Palynomorps (mapping mode, cored well A1-61)
Key a
LA-ICPMS measurements
(Quantitative scale on axis at left handside)
host rock
Tof-SIMS measurements
(Qualitative scale on axis at right hand side)
Chitinozoan Bi+
Scolecodont Bi+
Acritarch Bi+
Leiosphere Bi+
Scolecodont Bi3+
Chitinozoan Bi3+
Leiosphere Bi3+
Acritarch Bi3+
Spore Bi3+
Spore/algal cluster Bi3+
Arthropod Bi3+
Graptolite Bi3+
ppm (LA - ICPMS)
normalised # of counts at Tof-SIMS detector
Core depth (m)
Core 19 (teratologic acritarchs)
Core 14 (teratologic chitinozoans & acritarchs)
ToF-SIMS Ca+ data from Palynomorps (focussed beam mode - 133 to 200 sec, cored well A1-61)
Key b
Tof-SIMS measurements
(Qualitative scale on axis at right hand side)
Scolecodont (Bi3+)
Chitinozoan (Bi3+)
Acritarch (Bi3+)
Spore (Bi3+)
Underestimated values (saturation at detector)
Average of chitinozoans
ToF-SIMS Ca+ data from Palynomorps (mapping mode, core 14 : 2129,7 - 2125 m)
Key c
LA-ICPMS measurements
(Quantitative scale on axis at left handside)
host rock
Tof-SIMS measurements
(Qualitative scale on axis at right hand side)
Symbols ✚/●: see key a
ToF-SIMS Ca+ data from Palynomorps (focussed beam mode - 133 to 200 sec, core 14 : 2129,7 - 2125 m)
Key d
Tof-SIMS measurements
(Qualitative scale on axis at right hand side)
Symbols ✚/●: see key b
Number of teratologic chitinozoans per 1000 specimens counted
(from: Jaglin & Paris 1992)
(Quantitative scale on left hand side axis)
Teratologic specimens of chitinozoans observed during this study
Number of teratologic chitinozoans per 1000 specimens (from: Jaglin & Paris 1992)
interval with teratologic chitinozoans (Jaglin & Paris 1992 + this study)

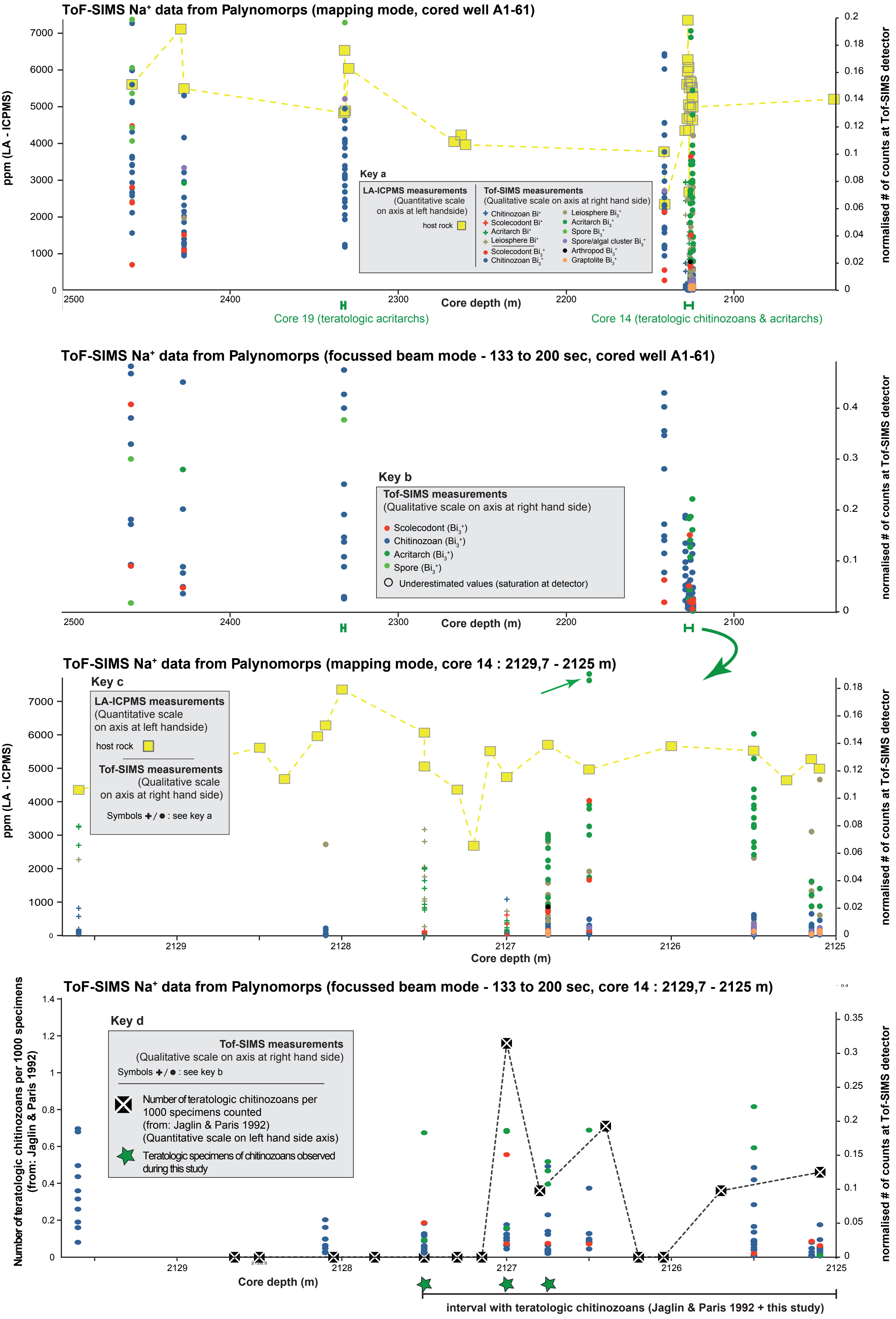

ToF-SIMS Na+ data from Palynomorps (mapping mode, cored well A1-61)
ppm (LA - ICPMS)
normalised # of counts at Tof-SIMS detector
Core depth (m)
Core 19 (teratologic acritarchs)
Core 14 (teratologic chitinozoans & acritarchs)
Key a
LA-ICPMS measurements
(Quantitative scale on axis at left handside)
host rock
Tof-SIMS measurements
(Qualitative scale on axis at right hand side)
Chitinozoan Bi+
Scolecodont Bi+
Acritarch Bi+
Leiosphere Bi+
Scolecodont Bi3+
Chitinozoan Bi3+
Leiosphere Bi3+
Acritarch Bi3+
Spore Bi3+
Spore/algal cluster Bi3+
Arthropod Bi3+
Graptolite Bi3+
ToF-SIMS Na+ data from Palynomorps (focussed beam mode - 133 to 200 sec, cored well A1-61)
Key b
Tof-SIMS measurements
(Qualitative scale on axis at right hand side)
Scolecodont (Bi3+)
Chitinozoan (Bi3+)
Acritarch (Bi3+)
Spore (Bi3+)
Underestimated values (saturation at detector)
ToF-SIMS Na+ data from Palynomorps (mapping mode, core 14 : 2129,7 - 2125 m)
Key c
LA-ICPMS measurements
(Quantitative scale on axis at left handside)
host rock
Tof-SIMS measurements
(Qualitative scale on axis at right hand side)
Symbols ✚/●: see key a
ToF-SIMS Na+ data from Palynomorps (focussed beam mode - 133 to 200 sec, core 14 : 2129,7 - 2125 m)
Number of teratologic chitinozoans per 1000 specimens (from: Jaglin & Paris 1992)
Key d
Tof-SIMS measurements
(Qualitative scale on axis at right hand side)
Symbols ✚/●: see key b
Number of teratologic chitinozoans per 1000 specimens counted
(from: Jaglin & Paris 1992)
(Quantitative scale on left hand side axis)
Teratologic specimens of chitinozoans observed during this study
interval with teratologic chitinozoans (Jaglin & Paris 1992 + this study)

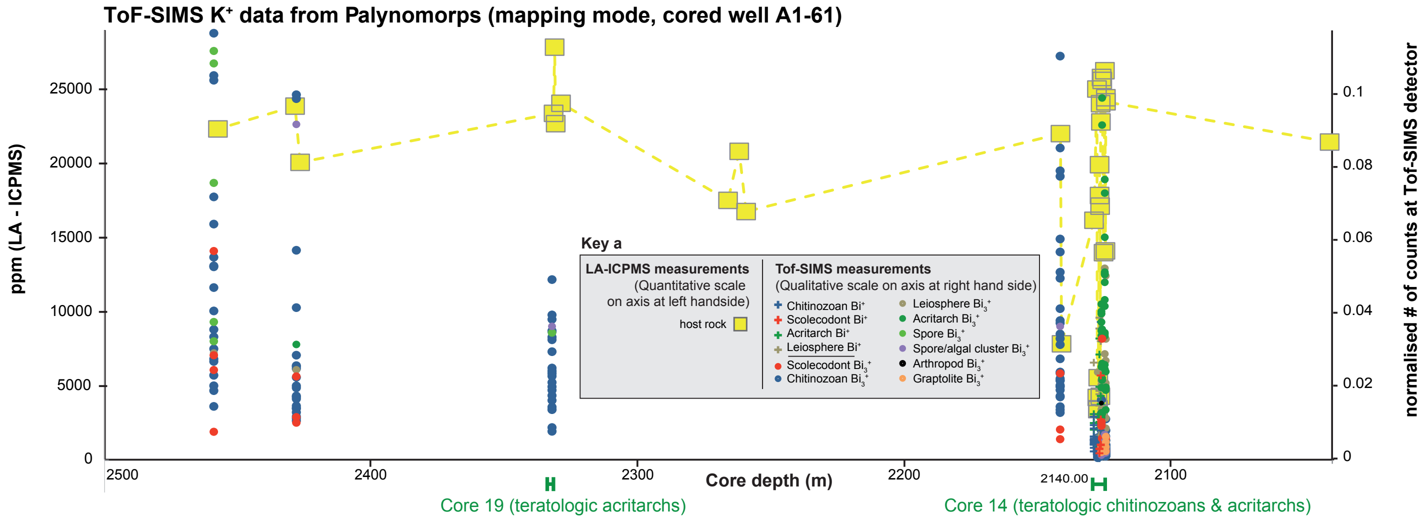
ToF-SIMS K+ data from Palynomorps (mapping mode, cored well A1-61)
ppm (LA - ICPMS)
25000
20000
15000
10000
5000
0
normalised # of counts at Tof-SIMS detector
0.1
0.08
0.06
0.04
0.02
0
Key a
LA-ICPMS measurements
(Quantitative scale on axis at left handside)
host rock
Tof-SIMS measurements
(Qualitative scale on axis at right hand side)
Chitinozoan Bi+
Scolecodont Bi+
Acritarch Bi+
Leiosphere Bi+
Scolecodont Bi3+
Chitinozoan Bi3+
Leiosphere Bi3+
Acritarch Bi3+
Spore Bi3+
Spore/algal cluster Bi3+
Arthropod Bi3+
Graptolite Bi3+
2500
2400
2300
Core depth (m)
2200
2140.00
2100
Core 19 (teratologic acritarchs)
Core 14 (teratologic chitinozoans & acritarchs)

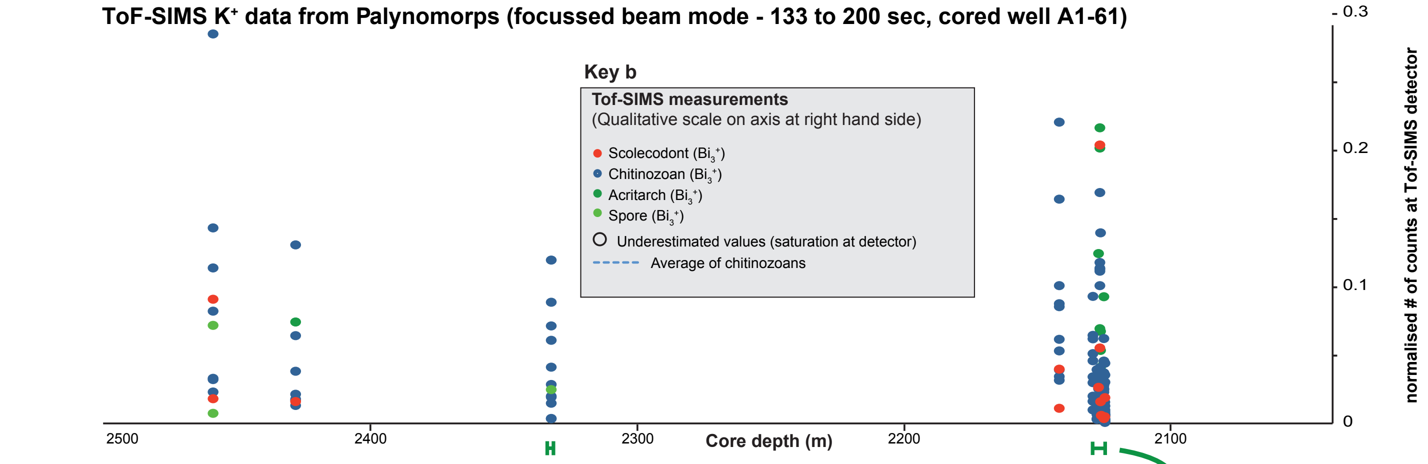
ToF-SIMS K+ data from Palynomorps (focussed beam mode - 133 to 200 sec, cored well A1-61)
0.3
0.2
0.1
0
normalised # of counts at Tof-SIMS detector
Key b
Tof-SIMS measurements
(Qualitative scale on axis at right hand side)
Scolecodont (Bi3+)
Chitinozoan (Bi3+)
Acritarch (Bi3+)
Spore (Bi3+)
Underestimated values (saturation at detector)
Average of chitinozoans
2500
2400
2300
Core depth (m)
2200
2100

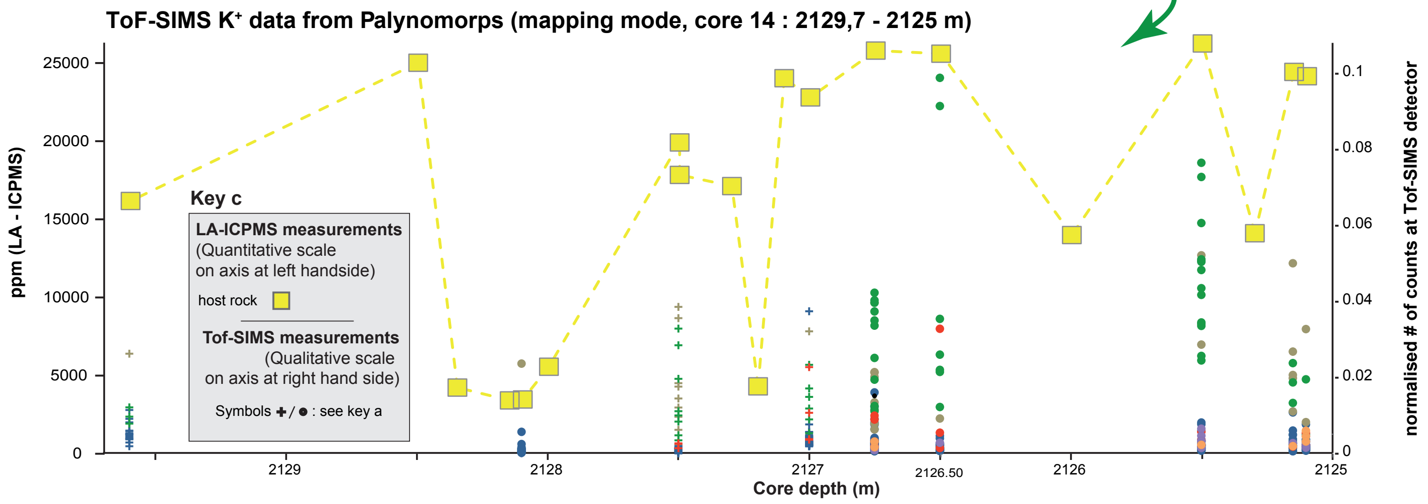
ToF-SIMS K+ data from Palynomorps (mapping mode, core 14 : 2129,7 - 2125 m)
ppm (LA - ICPMS)
25000
20000
15000
10000
5000
0
normalised # of counts at Tof-SIMS detector
0.1
0.08
0.06
0.04
0.02
0
Key c
LA-ICPMS measurements
(Quantitative scale
on axis at left handside)
host rock
Tof-SIMS measurements
(Qualitative scale
on axis at right hand side)
Symbols +/●: see key a
2129
2128
2127
2126.50
2126
2125
Core depth (m)

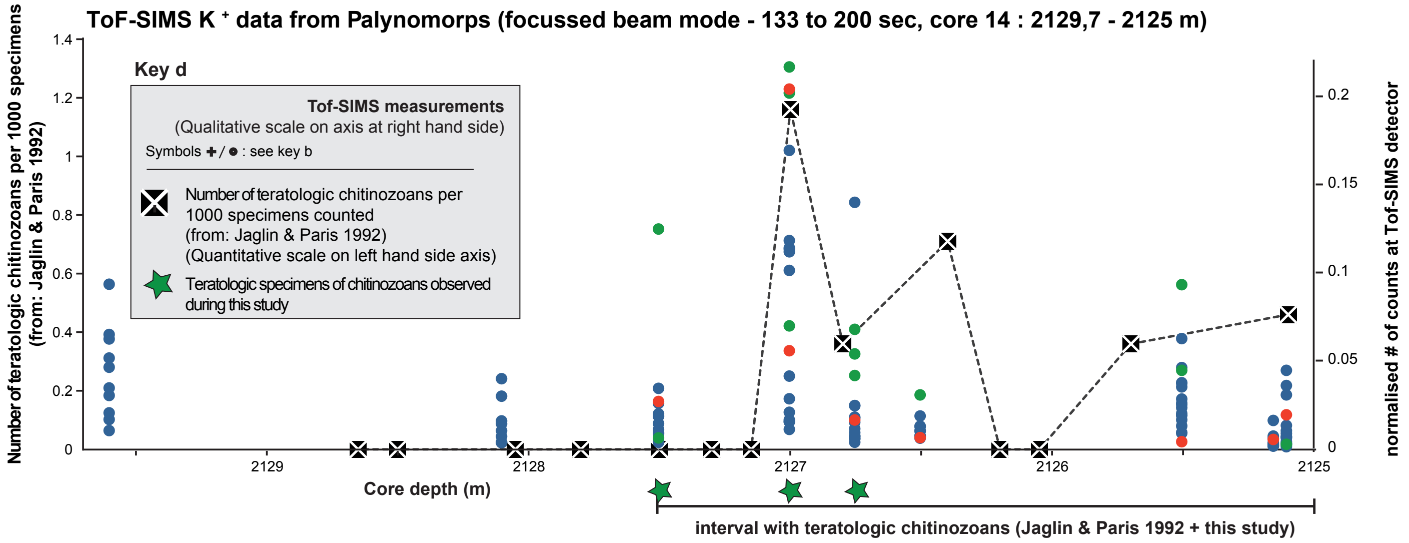
ToF-SIMS K + data from Palynomorps (focussed beam mode - 133 to 200 sec, core 14 : 2129,7 - 2125 m)
Number of teratologic chitinozoans per 1000 specimens
(from: Jaglin & Paris 1992)
1.4
1.2
1
0.8
0.6
0.4
0.2
0
normalised # of counts at Tof-SIMS detector
0.2
0.15
0.1
0.05
0
Key d
Tof-SIMS measurements
(Qualitative scale on axis at right hand side)
Symbols +/●: see key b
Number of teratologic chitinozoans per
1000 specimens counted
(from: Jaglin & Paris 1992)
(Quantitative scale on left hand side axis)
Teratologic specimens of chitinozoans observed
during this study
2129
2128
2127
2126
2125
Core depth (m)
interval with teratologic chitinozoans (Jaglin & Paris 1992 + this study)

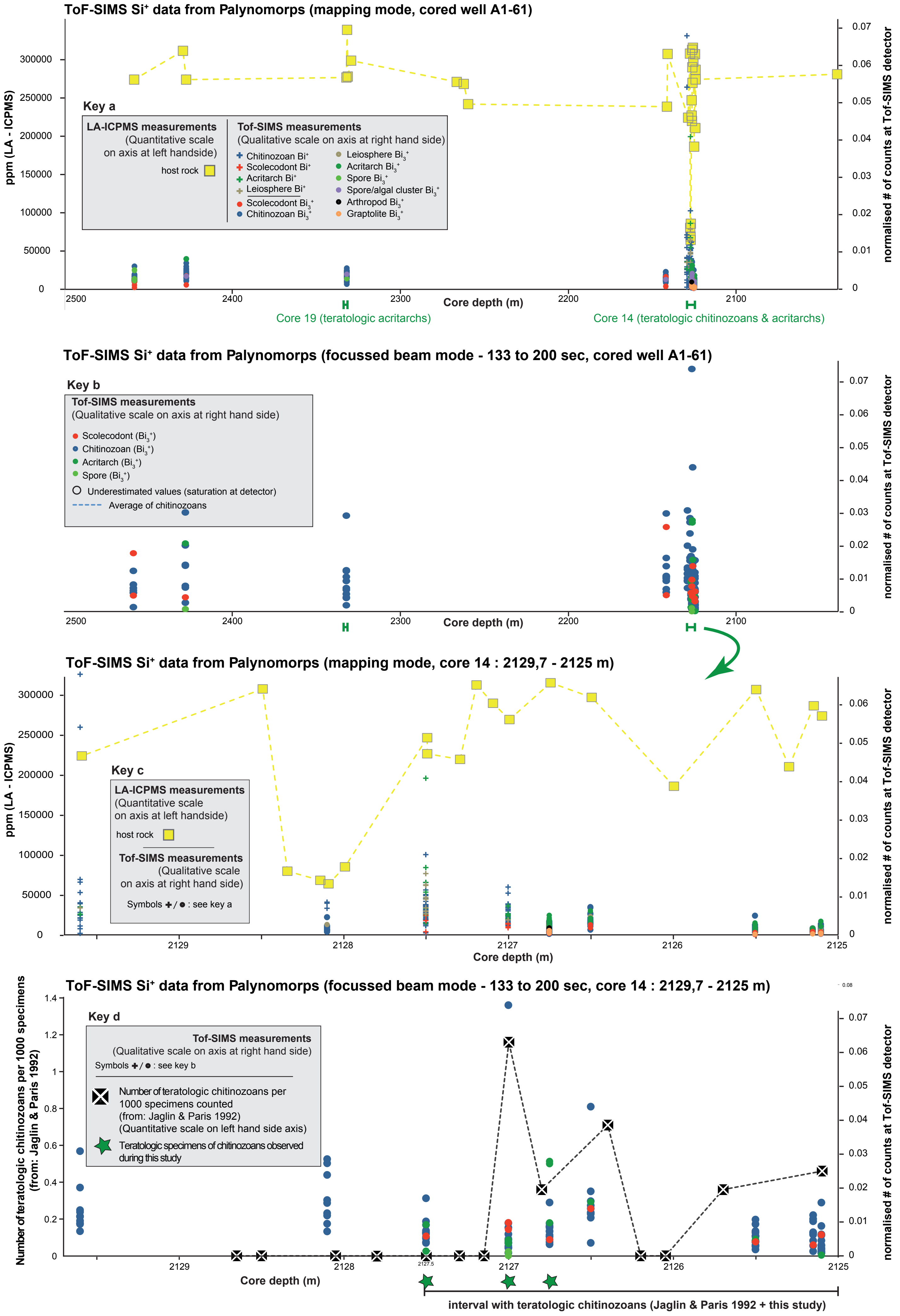

ToF-SIMS Si+ data from Palynomorps (mapping mode, cored well A1-61)
ppm (LA - ICPMS)
300000
250000
200000
150000
100000
50000
0
normalised # of counts at Tof-SIMS detector
0.07
0.06
0.05
0.04
0.03
0.02
0.01
0
Key a
LA-ICPMS measurements
(Quantitative scale on axis at left handside)
host rock
Tof-SIMS measurements
(Qualitative scale on axis at right hand side)
Chitinozoan Bi+
Scolecodont Bi+
Acritarch Bi+
Leiosphere Bi+
Scolecodont Bi3+
Chitinozoan Bi3+
Leiosphere Bi3+
Acritarch Bi3+
Spore Bi3+
Spore/algal cluster Bi3+
Arthropod Bi3+
Graptolite Bi3+
2500
2400
2300
Core depth (m)
2200
2100
Core 19 (teratologic acritarchs)
Core 14 (teratologic chitinozoans & acritarchs)
ToF-SIMS Si+ data from Palynomorps (focussed beam mode - 133 to 200 sec, cored well A1-61)
Key b
Tof-SIMS measurements
(Qualitative scale on axis at right hand side)
Scolecodont (Bi3+)
Chitinozoan (Bi3+)
Acritarch (Bi3+)
Spore (Bi3+)
Underestimated values (saturation at detector)
Average of chitinozoans
normalised # of counts at Tof-SIMS detector
0.07
0.06
0.05
0.04
0.03
0.02
0.01
0
2500
2400
2300
Core depth (m)
2200
2100
ToF-SIMS Si+ data from Palynomorps (mapping mode, core 14 : 2129,7 - 2125 m)
ppm (LA - ICPMS)
300000
250000
200000
150000
100000
50000
0
Key c
LA-ICPMS measurements
(Quantitative scale
on axis at left handside)
host rock
Tof-SIMS measurements
(Qualitative scale
on axis at right hand side)
Symbols ✚ / ● : see key a
normalised # of counts at Tof-SIMS detector
0.06
0.05
0.04
0.03
0.02
0.01
0
2129
2128
2127
2126
2125
Core depth (m)
ToF-SIMS Si+ data from Palynomorps (focussed beam mode - 133 to 200 sec, core 14 : 2129,7 - 2125 m)
Number of teratologic chitinozoans per 1000 specimens
(from: Jaglin & Paris 1992)
1.4
1.2
1
0.8
0.6
0.4
0.2
0
Key d
Tof-SIMS measurements
(Qualitative scale on axis at right hand side)
Symbols ✚ / ● : see key b
Number of teratologic chitinozoans per 1000 specimens counted
(from: Jaglin & Paris 1992)
(Quantitative scale on left hand side axis)
Teratologic specimens of chitinozoans observed during this study
0.08
0.07
0.06
0.05
0.04
0.03
0.02
0.01
0
normalised # of counts at Tof-SIMS detector
2129
2128
2127.5
2127
2126
2125
Core depth (m)
interval with teratologic chitinozoans (Jaglin & Paris 1992 + this study)

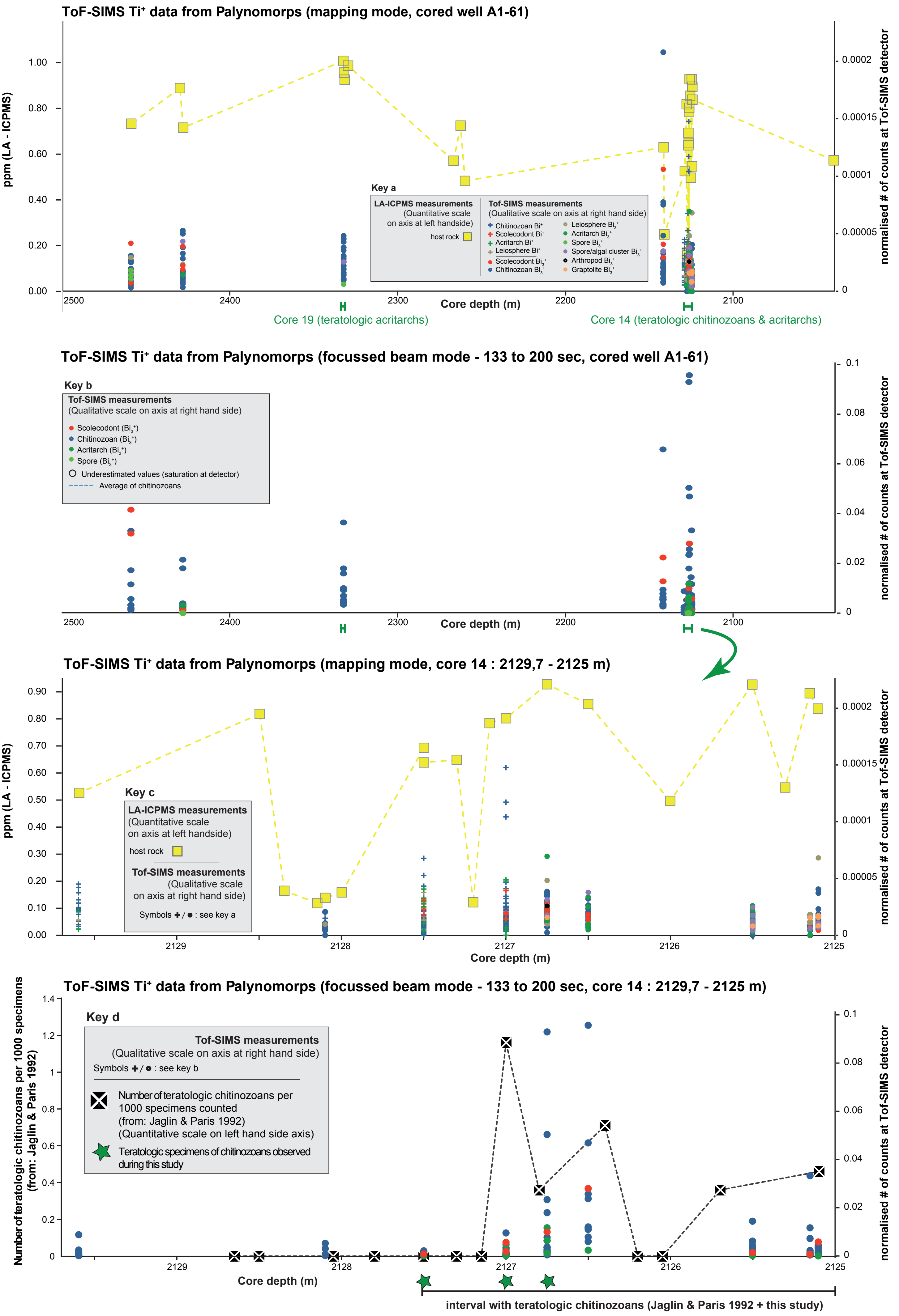

ToF-SIMS Ti+ data from Palynomorps (mapping mode, cored well A1-61)
ppm (LA - ICPMS)
normalised # of counts at Tof-SIMS detector
Key a
LA-ICPMS measurements
(Quantitative scale on axis at left handside)
host rock
Tof-SIMS measurements
(Qualitative scale on axis at right hand side)
Chitinozoan Bi+
Scolecodont Bi+
Acritarch Bi+
Leiosphere Bi+
Scolecodont Bi3+
Chitinozoan Bi3+
Leiosphere Bi3+
Acritarch Bi3+
Spore Bi3+
Spore/algal cluster Bi3+
Arthropod Bi3+
Graptolite Bi3+
Core depth (m)
Core 19 (teratologic acritarchs)
Core 14 (teratologic chitinozoans & acritarchs)
ToF-SIMS Ti+ data from Palynomorps (focussed beam mode - 133 to 200 sec, cored well A1-61)
Key b
Tof-SIMS measurements
(Qualitative scale on axis at right hand side)
Scolecodont (Bi3+)
Chitinozoan (Bi3+)
Acritarch (Bi3+)
Spore (Bi3+)
Underestimated values (saturation at detector)
Average of chitinozoans
Core depth (m)
ToF-SIMS Ti+ data from Palynomorps (mapping mode, core 14 : 2129,7 - 2125 m)
Key c
LA-ICPMS measurements
(Quantitative scale on axis at left handside)
host rock
Tof-SIMS measurements
(Qualitative scale on axis at right hand side)
Symbols ✚ / ● : see key a
Core depth (m)
ToF-SIMS Ti+ data from Palynomorps (focussed beam mode - 133 to 200 sec, core 14 : 2129,7 - 2125 m)
Number of teratologic chitinozoans per 1000 specimens (from: Jaglin & Paris 1992)
Key d
Tof-SIMS measurements
(Qualitative scale on axis at right hand side)
Symbols ✚ / ● : see key b
Number of teratologic chitinozoans per 1000 specimens counted
(from: Jaglin & Paris 1992)
(Quantitative scale on left hand side axis)
Teratologic specimens of chitinozoans observed during this study
Core depth (m)
interval with teratologic chitinozoans (Jaglin & Paris 1992 + this study)

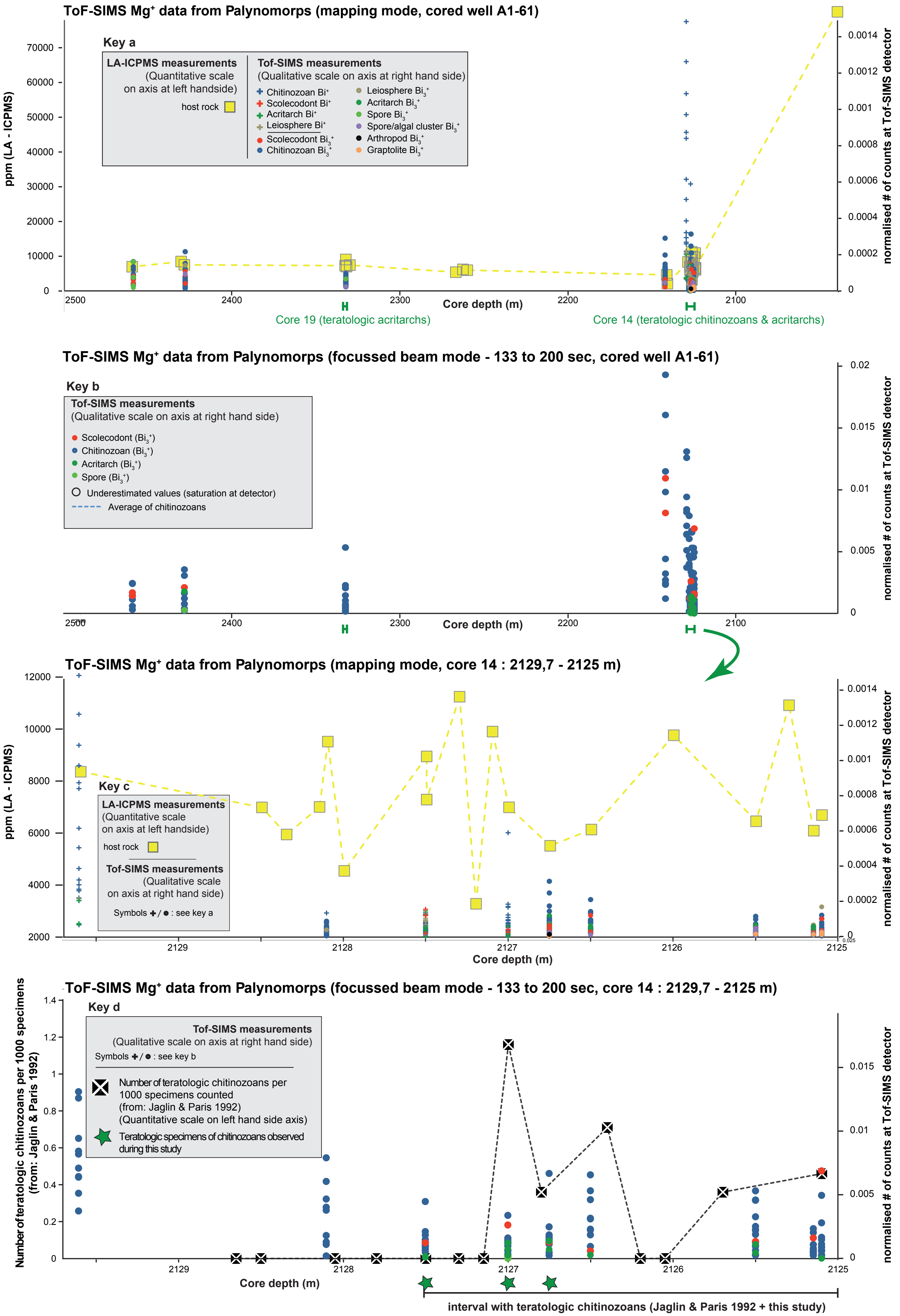
ToF-SIMS Mg+ data from Palynomorps (mapping mode, cored well A1-61)
Key a
LA-ICPMS measurements
(Quantitative scale on axis at left handside)
host rock
Tof-SIMS measurements
(Qualitative scale on axis at right hand side)
Chitinozoan Bi+
Scolecodont Bi+
Acritarch Bi+
Leiosphere Bi+
Scolecodont Bi3+
Chitinozoan Bi3+
Leiosphere Bi3+
Acritarch Bi3+
Spore Bi3+
Spore/algal cluster Bi3+
Arthropod Bi3+
Graptolite Bi3+
ppm (LA - ICPMS)
normalised # of counts at Tof-SIMS detector
Core depth (m)
Core 19 (teratologic acritarchs)
Core 14 (teratologic chitinozoans & acritarchs)
ToF-SIMS Mg+ data from Palynomorps (focussed beam mode - 133 to 200 sec, cored well A1-61)
Key b
Tof-SIMS measurements
(Qualitative scale on axis at right hand side)
Scolecodont (Bi3+)
Chitinozoan (Bi3+)
Acritarch (Bi3+)
Spore (Bi3+)
Underestimated values (saturation at detector)
Average of chitinozoans
ToF-SIMS Mg+ data from Palynomorps (mapping mode, core 14 : 2129,7 - 2125 m)
Key c
LA-ICPMS measurements
(Quantitative scale on axis at left handside)
host rock
Tof-SIMS measurements
(Qualitative scale on axis at right hand side)
Symbols + / ● : see key a
ToF-SIMS Mg+ data from Palynomorps (focussed beam mode - 133 to 200 sec, core 14 : 2129,7 - 2125 m)
Key d
Tof-SIMS measurements
(Qualitative scale on axis at right hand side)
Symbols + / ● : see key b
Number of teratologic chitinozoans per 1000 specimens counted (from: Jaglin & Paris 1992) (Quantitative scale on left hand side axis)
Teratologic specimens of chitinozoans observed during this study
Number of teratologic chitinozoans per 1000 specimens (from: Jaglin & Paris 1992)
interval with teratologic chitinozoans (Jaglin & Paris 1992 + this study)
